# Imaging‐Based Molecular Characterization of Adult‐Type Diffuse Glioma Using Diffusion and Perfusion MRI in Pre‐ and Post‐Treatment Stage Considering Spatial and Temporal Heterogeneity

**DOI:** 10.1002/jmri.29781

**Published:** 2025-04-08

**Authors:** Yun Hwa Roh, E‐Nae Cheong, Ji Eun Park, Yangsean Choi, Seung Chai Jung, Sang Woo Song, Young‐Hoon Kim, Chang‐Ki Hong, Jeong Hoon Kim, Ho Sung Kim

**Affiliations:** ^1^ Department of Radiology, Samsung Medical Center Sungkyunkwan University School of Medicine Seoul Republic of Korea; ^2^ Department of Radiology and Research Institute of Radiology University of Ulsan College of Medicine, Asan Medical Center Seoul Republic of Korea; ^3^ Department of Neurosurgery University of Ulsan College of Medicine, Asan Medical Center Seoul Republic of Korea

**Keywords:** characterization, EGFR, glioma, heterogeneity, IDH

## Abstract

**Background:**

Imaging‐based molecular characterization is important for identifying treatment targets in adult‐type diffuse gliomas.

**Purpose:**

To assess isocitrate dehydrogenase (IDH) mutation and epidermal growth factor receptor (EGFR) amplification status in primary and recurrent gliomas using diffusion and perfusion MRI, addressing spatial and temporal heterogeneity.

**Study Type:**

Retrospective.

**Subjects:**

Three‐hundred and twelve newly diagnosed (cross‐sectional set, 57.9 ± 13.2 years, 52.2% male, 235 IDH‐wildtype, 71 EGFR‐amplified) and 38 recurrent (longitudinal set, 53.1 ± 13.4 years, 44.7% male, 30 IDH‐wildtype, 13 EGFR‐amplified) adult‐type diffuse glioma patients.

**Field Strength/Sequence:**

3.0T; diffusion weighted and dynamic susceptibility contrast‐perfusion weighted imaging.

**Assessment:**

Radiomics features from contrast‐enhancing tumors (CET) and non‐enhancing lesions (NEL) were extracted from apparent diffusion coefficient and perfusion maps. Spatial heterogeneity was assessed using intersection and Bhattacharyya distance between CET and NEL. Stable imaging features were identified in patients with unchanged genetic profiles in the longitudinal set. The “best model,” using features from the cross‐sectional set (*n* = 312), and the “concordant model,” using stable features identified in the longitudinal set (*n* = 38), were constructed using the LASSO for IDH and EGFR status.

**Statistical Tests:**

The area under the receiver‐operating‐characteristic curve (AUC).

**Results:**

For IDH mutations, both best and concordant models demonstrated high AUCs in the cross‐sectional set (0.936; 95% confidence interval [CI]: 0.903–0.969 and 0.964 [0.943–0.986], respectively). Only the concordant model maintained strong performance in recurrent tumors (AUC, 0.919 vs. 0.656). For EGFR amplification in IDH‐wildtype, the best and concordant models showed AUCs of 0.821 (95% CI: 0.761–0.881) and 0.746 (95% CI: 0.675–0.817) in newly diagnosed gliomas, but poor performance in recurrent tumors with AUCs of 0.503 (95% CI: 0.34–0.665) and 0.518 (95% CI: 0.357–0.678).

**Data Conclusion:**

Diffusion and perfusion MRI characterized IDH status in both newly diagnosed and recurrent gliomas, but showed limited diagnostic performance for EGFR, especially for recurrent tumors.

**Evidence Level:**

3

**Technical Efficacy:**

Stage 3

**Plain Language Summary**

- Noninvasive imaging‐based characterization of molecular status is important for identifying potential treatment targets in adult‐type diffuse gliomas.
- This study aimed to assess IDH mutation and EGFR amplification in primary and recurrent gliomas using radiomics features derived from perfusion and diffusion MRI.
- It focused on spatial heterogeneity, representing molecular variability within the tumor, and temporal heterogeneity, reflecting dynamic changes in the tumor over time.
- Radiomics models reliably characterized IDH mutation in both newly diagnosed and recurrent gliomas but showed limited accuracy in EGFR amplification, particularly in recurrent tumors.

## 1 Introduction

The World Health Organization (WHO) classification has increasingly incorporated molecular analysis stratification into adult‐type diffuse gliomas [1]. Molecular subtyping plays an important role in prognosis and identification of potential targets for effective therapies. Isocitrate dehydrogenase (IDH) mutations occur early in tumor development, particularly in low‐grade gliomas, and are associated with improved survival outcomes compared with IDH‐wildtype glioblastomas [2]. Epidermal growth factor receptor (EGFR) amplification and mutations are frequently observed in IDH‐wildtype glioblastomas, which are associated with poor prognosis and represent potential targets for molecular therapies [3, 4].

Imaging‐based molecular characterizations require careful consideration of both spatial and temporal heterogeneity [5]. Spatial heterogeneity refers to the variability in molecular and genetic profiles across different regions of the tumor, whereas temporal heterogeneity reflects dynamic changes over time and is influenced by tumor evolution or treatment response. For example, IDH mutations are ubiquitously expressed and remain stable over time [6], whereas EGFR amplification demonstrates marked spatial variability between different tumor regions and frequently occurs and disappears over time [7, 8]. Addressing these heterogeneities is important for developing robust imaging‐based molecular assessment models capable of guiding the noninvasive prediction of prognosis and targeted therapies.

Diffusion‐ and perfusion‐weighted MRI has been employed to analyze spatial heterogeneity in gliomas. Lower apparent diffusion coefficient (ADC) values, indicating higher cellularity, are associated with IDH‐wildtype and EGFR amplification in gliomas [9, 10, 11]. In IDH‐mutant gliomas, relative cerebral blood volume (rCBV) values are typically lower and more uniformly distributed, likely due to the inhibitory effects of 2‐hydroxyglutarate on hypoxia and angiogenesis as well as the ubiquitous expression of IDH mutations across tumor cells [5, 12]. Meanwhile, EGFR‐amplified tumors exhibit higher rCBV [13] and elevated relative oxygen extraction fraction (OEF) [14], indicating increased tumor cell invasion and angiogenesis. Additionally, EGFRvIII‐positive tumors exhibit a more isotropic rCBV distribution across enhancing and non‐enhancing tumor regions [15].

Despite advances in imaging‐based spatial analysis, longitudinal assessment of molecular changes remains a challenge. Most previous studies have been limited to cross‐sectional data at single time points, typically in patients with newly diagnosed tumors [11, 13, 16, 17]. However, adult‐type diffuse gliomas almost invariably recur and undergo molecular evolution, further complicating the spatial and temporal heterogeneity [18]. EGFR amplification in recurrent tumors differs from that in primary glioblastomas [7, 8], with a concordance correlation coefficient of 0.65 [8]. This highlights the necessity of considering temporal heterogeneity when assessing molecular changes, particularly those related to EGFR.

Thus the aim of this study was to assess IDH mutations and EGFR amplification status in primary and recurrent gliomas using diffusion and perfusion MRI. Specifically, the aim was to analyze imaging features and distribution patterns of enhancing and non‐enhancing regions to address spatial heterogeneity and to identify imaging features consistent across primary and recurrent tumors to account for temporal heterogeneity.

## 2 Materials and Methods

### 2.1 Study Population

This retrospective study was approved by the Institutional Review Board of Asan Medical Center, and the requirement for informed consent was waived (IRB no. 2023‐0525).

For the cross‐sectional set, the database of the Department of Radiology and Neurosurgery at our tertiary center between March 2017 and March 2023 was reviewed, and 401 consecutive patients with pathologically confirmed (on surgical resection or biopsy) adult‐type diffuse gliomas (Grades 2, 3, and 4) according to the WHO 2021 classification of central nervous system tumors were identified. The exclusion criteria were as follows [1]: Non‐availability of IDH mutation or EGFR amplification status (*n* = 43); [2] missing preoperative multi‐parametric MRI (*n* = 38); and [3] imaging that could not be processed because of technical reasons (*n* = 8). Finally, 312 patients (57.9 ± 13.2 years; range, 23–85; 164 men [52.6%]) were included.

**FIGURE 1:**
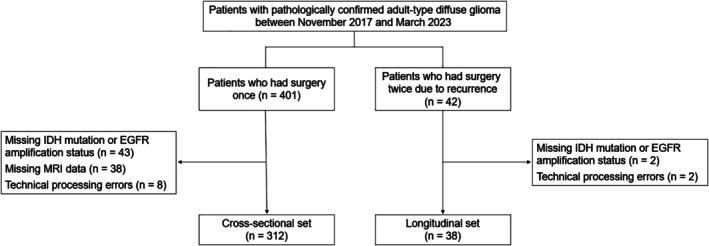
Patient selection process. EGFR, epidermal growth factor receptor; IDH, isocitrate dehydrogenase.

For the longitudinal set, 42 patients who had undergone two surgeries were included: An initial surgery following the diagnosis of adult‐type diffuse glioma and a subsequent surgery due to recurrence, both within the study period. Following the exclusion of patients with missing IDH mutations or EGFR amplification status in either surgery (*n* = 2) or due to technical processing errors (*n* = 2), 38 patients (53.1 ± 13.4 years; range, 25–75; 17 men [44.7%]) with 76 specimens were included in the longitudinal set. Preoperative MRI scans obtained before the first surgery (referred to as initial MRI) and preoperative MRI scans obtained before the second surgery (referred to as recurrence MRI) were included for each patient. A flow diagram of the patient selection process is shown in Figure 1.

### 2.2 Reference Standard of IDH Mutation and EGFR Amplification

The histopathological diagnoses were based on the 2021 WHO classification system [1]. Immunohistochemical analysis and next generation sequencing were performed to detect IDH1 R132H mutations. IDH mutations were assessed at the R132 and R172 codons in IDH1 and IDH2, respectively.

Targeted next‐generation sequencing was performed using the Illumina NextSeq 500Dx panel. For copy number analysis, copy number variation amplification detections were performed for EGFR (EGFR+, gain vs. balanced). EGFR genes showing a > 2‐fold change relative to the average level were considered amplified.

### 2.3 Imaging Acquisition

All MRI studies were performed using a 3‐T scanner (Ingenia 3.0T CX; Philips Healthcare, Best, the Netherlands). Both cross‐sectional and longitudinal sets followed the same MRI protocol, including T2‐weighted fast spin‐echo imaging, fluid‐attenuated inversion recovery (FLAIR) fast spin‐echo imaging, three‐dimensional pre‐contrast and contrast‐enhanced T1‐weighted gradient‐echo imaging (CE‐T1WI), diffusion weighted fast spin‐echo imaging (DWI), and dynamic susceptibility contrast (DSC) perfusion gradient‐echo MRI. The detailed MRI acquisition parameters are provided in Supporting Information S1. DWI was obtained in three orthogonal directions using the following parameters: repetition time (TR)/echo time (TE), 3000/56 ms; diffusion gradient encoding, *b* = 0, 1000 s/mm^2^; field of view (FOV), 25 cm; slice thickness/gap, 5/2 mm; matrix, 256 × 256; and acquisition time, 39 s. CE‐T1WI was obtained with a higher‐resolution three‐dimensional volume image using the following parameters: TR/TE, 9.8/4.6 ms; flip angle, 10°; FOV, 256 mm; matrix, 512 × 512; and slice thickness, 0.5 mm with no gap. DSC‐MRI was performed using a gradient‐echo echo‐planar imaging sequence with TR/TE, 1425/30 ms; flip angle, 60°; FOV, 23 cm; slice thickness/gap, 4/2 mm; matrix, 128 × 128; and total acquisition time, 2 min 55 s. All patients were administered a preload of 0.01 mmol/kg gadoterate meglumine (Dotarem; Guerbet, Paris, France), followed by a dynamic bolus consisting of a standard dose of 0.1 mmol/kg gadoterate meglumine delivered at a rate of 4 mL/s using an MRI‐compatible power injector (Spectris; Medrad, Pittsburgh, PA). Subsequently, a 20‐mL bolus of saline was injected at the same rate.

### 2.4 Image Processing

For tumor segmentation, skull stripping was performed using the three‐dimensional CE‐T1WI and FLAIR images with an algorithm optimized to manage heterogeneous MRI data, accounting for variations caused by different pathologies or post‐treatment changes (https://github.com/MIC‐DKFZ/HD‐BET). Tumor segmentation masks for contrast‐enhancing tumor (CET) and non‐enhancing lesion (NEL) were created on three‐dimensional CE‐T1WI and FLAIR images using a 3D UNet‐based method (https://github.com/MIC‐DKFZ/nnUNet) [19] and the PyTorch package version 1.1 in Python 3.7 (www.python.org). The segmentation approach was based on a model from the BraTS21 challenge framework, which leverages the robust training and generalization capabilities of nnU‐Net [20]. The BraTS21 dataset, comprising 1251 skull‐stripped multi‐parametric MRI scans across four modalities (FLAIR, T1, CE‐T1WI, and T2), provided the foundation for this model. Three‐dimensional CE‐T1WI and FLAIR data were used as input imaging modalities to generate the segmentation of contrast‐enhancing lesions, NEL, and necrosis. The segmentation model was trained on data from 455 patients with brain tumors and validated using 2034 MRI scans from 532 patients as part of the EORTC‐26101 study. Technical and clinical validation of this method has been reported previously [20, 21, 22]. A neuroradiologist (Ji Eun Park, with 9 years of experience in neuro‐oncologic imaging) reviewed and validated all segmented results.

The three‐dimensional CE‐T1WI, T1 co‐registered FLAIR, and segmentation masks were co‐registered with the mean DSC. Similarly, the ADC map calculated using DWI was co‐registered with the mean DSC. This was important for aligning the segmentation masks with the diffusion and perfusion maps for spatial mapping.

For DSC analysis, all standard perfusion maps, along with parametric maps related to microvascular and oxygen metabolism, were automatically generated using Cercare Medical Neurosuite (Cercare Medical ApS, release 2021‐03‐02‐02), as previously described [23, 24]. The arterial input function (AIF) was automatically identified using cluster analysis techniques, and its deconvolution was performed using time‐insensitive block‐circulant singular value decomposition [25]. AIF was provided by Cercare software, and a researcher (Roh Yoon Wha, with 3 years of experience in neuro‐oncologic imaging.) checked its validity. The rCBV and relative cerebral blood flow (rCBF) were calculated by fitting a gamma variate‐based vascular model to the measured R2* curve. Capillary transit time heterogeneity (CTH) maps were obtained by computing the standard deviation of the gamma distribution. Coefficient of variation (COV) maps were calculated by dividing CTH by the mean transit time (MTT). The OEF and cerebral metabolic rate of oxygen (CMRO_2_) maps were created using the software. The rCBV and rCBF values were normalized to nCBV and nCBF in normal‐appearing white matter, respectively, as provided by the software. The perfusion maps were resampled to match the iso‐voxel three‐dimensional CE‐T1WI using rigid transformations with 6° of freedom in SPM12 (www.fil.ion.ucl.ac.uk/spm/). Perfusion‐, microvasculature‐, and oxygen‐related values were calculated using the CET volume.

### 2.5 Feature Extraction

To quantitatively characterize the tumor regions of interest (ROIs; CET and NEL) and account for their physiological background, a radiomics approach was employed using the open‐source Python Library PyRadiomics (version 3.0.1) [26]. Fourteen shape features were extracted from the two ROIs. Additionally, 93 radiomics features were extracted from each ROI across nine parametric maps (ADC, nCBV, nCBF, MTT, time to peak [TTP], CTH, COV, OEF, and CMRO_2_). These 93 features comprised 18 first‐order features, 24 Gray Level Co‐occurrence Matrix (GLCM) features, 16 Gray Level Run Length Matrix (GLRLM) features, 16 Gray Level Size Zone Matrix (GLSZM) features, 14 Gray Level Dependence Matrix (GLDM) features, and 5 Neighboring Gray Tone Difference Matrix (NGTDM) features. Detailed descriptions of the extracted radiomics features can be found in the PyRadiomics documentation available at https://pyradiomics.readthedocs.io/en/latest/features.html.

To account for the spatial heterogeneity between CET and NEL, the intersection and Bhattacharyya distances were used [15, 27]. These metrics quantify the differences (or similarities) between two histograms, H1 and H2: The mathematical equations for each method are as follows: The CET and NEL histograms are represented as H1 and H2, respectively.

$$D_{\text{intersection}}\;\left(H1,H2\right)=\sum \min\left(H1\left(i\right),H2\left(i\right)\right)$$

The intersection uses the minimum value across the corresponding bins to quantify the overlap and measures the common area under the two histograms. The value lies between zero and one. A high value (closer to 1) indicates a close match, whereas a low value (closer to 0) indicates a poor match.

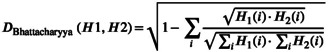




The Bhattacharyya distance uses the geometric mean and logarithm to quantify the overlap between the two distributions. The Bhattacharyya coefficient (before taking the negative logarithm) lies between 0 and 1, with a lower value indicating a better match. A value of 0 represents a perfect match (identical distribution), and a value of 1 represents an absolute non‐match.

For enhancing tumor and non‐enhancing tumor ROIs, 93 radiomics features were extracted for each of the nine parametric maps (ADC, nCBV, nCBF, MTT, TTP, CTH, COV, OEF, and CMRO_2_) across the two ROIs (CET and NEL). Fourteen shape features were extracted from each ROI. Two spatial heterogeneity features (intersection and Bhattacharyya distance) were then calculated for each parametric map, resulting in a final count of 1720 features per patient, using the following formula:

$$\begin{array}{c} \left(\text{Radiomics features}\;\times \;\text{parametric maps}\;\times \;\text{ROIs}\right)+\left(\text{shape features}\;\times \;\text{ROIs}\right)\\ +\left(\text{parametric maps}\;\times \;\text{spatial heterogeneity features}\right) \end{array}$$

Substituting the values: (93 × 9 × 2) + (14 × 2) + (9 × 2) = 1720. In total, 1720 features were extracted from each patient.

### 2.6 Statistical Analysis

#### 2.6.1 Power Analysis

The target distribution was EGFR amplification, reported in 20%–30% of glioblastomas [28], and this distribution was consistent with the longitudinal cohort in the current study. The null hypothesis assumed an area under the receiver operating characteristic curve (AUC) of 0.50, whereas alternative hypothesis targeted an AUC of 0.80 for the radiomics model in detecting EGFR amplification. The statistical parameters included a Type I error (*α*) of 0.05 and a Type II error (*β*) of 0.20, corresponding to a statistical power of 80% [29]. Based on these parameters, the required sample size was 36. Because the longitudinal cohort included 38 patients, the statistical power was deemed sufficient to ensure a robust analysis.

#### 2.6.2 Clinical Characteristics

Differences in patient and tumor characteristics between the cross‐sectional and longitudinal sets and imaging parameters according to the IDH mutation or EGFR amplification status were evaluated using independent samples *t‐*tests and chi‐square tests.

#### 2.6.3 Assessing Concordance of the Features Between Initial and Recurrence MRI in the Longitudinal Set

In the longitudinal dataset, patients with unchanged genetic profiles between the first and second surgeries were identified. We hypothesized that if the genetic profile remained stable, the corresponding MRI features would also remain stable over time. Temporally stable MRI features were defined as those showing no substantial variation based on concordance analysis between the initial MRI and the recurrence MRI in these patients.

Using this definition, the intraclass correlation coefficient (ICC) was calculated to quantify the concordance of radiomics features between the two MRI studies. Since the IDH mutation status did not change in any of the patients, the ICCs for the radiomics features between the initial MRI and recurrence MRI were calculated across all patients. Features with an ICC threshold > 0.5 were considered [30], which is a commonly used threshold for ICC, temporally stable, and selected for classification to diagnose IDH mutation.

Similarly, to identify temporally stable MRI features for EGFR amplification in IDH‐wildtype tumors, patients exhibiting a consistent EGFR amplification status (i.e., either amplification or no amplification) between the first and second surgeries were included. The ICC of the radiomics features on the initial and recurrence MRI scans for these patients was calculated. Features with an ICC > 0.5 were defined as temporally stable and selected for constructing a diagnostic model for EGFR amplification.

#### 2.6.4 Feature Selection and Model Construction

The least absolute shrinkage and selection operator (LASSO) logistic model was applied to identify significant features with nonzero coefficients within the two sets of stable features related to consistent genetic profiles. Linear predictors for IDH mutation for all patients and EGFR amplification for patients with IDH‐wildtype tumors were constructed using logistic regression, resulting in “concordant models.” Subsequently, LASSO was applied to the entire features within the cross‐sectional set to select significant predictors for genetic mutations. When a group of features is correlated with each other, LASSO tends to select only one of them [31]. This reduces false‐positive findings and subsequent errors. These models were referred to as “best models.” In summary, four models were developed: The “best model” and the “concordant model” for diagnosing IDH mutation and for diagnosing EGFR amplification in IDH‐wildtype tumors.

#### 2.6.5 Diagnostic Performance

The diagnostic performance of the best and concordant models using cross‐sectional and longitudinal sets was measured using the AUC. The optimal thresholds of AUCs were determined by maximizing the sum of the sensitivity and specificity values calculated to predict the diagnosis using the Youden index. The accuracy, sensitivity, and specificity of optimal thresholds were calculated.

For all statistical analyses, two‐sided *p* values < 0.05 were considered statistically significant. All statistical analyses were performed using R statistical software (version 4.1.3, Vienna, Austria).

## 3 Results

### 3.1 Baseline Characteristics

Table 1 summarizes the baseline clinical characteristics of the 312 patients in the cross‐sectional set (77 IDH‐mutant, 235 IDH‐wildtype) and 38 patients in the longitudinal set (8 IDH‐mutant, 30 IDH‐wildtype). Patients in the longitudinal set were significantly younger than those in the cross‐sectional set, with no significant differences in sex (*p* = 0.459), proportion of histopathologic grade (*p* = 0.393), and IDH mutations (*p* = 0.77).

**TABLE 1:** Clinical characteristics of patients.

	Cross‐sectional set (*n* = 312)	Longitudinal set (*n* = 38)	*p*
Age, years (mean ± SD)	57.9 ± 13.2	53.1 ± 13.4	0.034*
Sex, male (%)	164 (52.6)	17 (44.7)	0.459
Histopathologic grade			
Grade 2 or 3	82 (26.3)	7 (18.4)	0.393
Grade 4	230 (73.7)	31 (81.6)	
WHO 2021 classification			
Oligodendroglioma, IDH‐mutant, 1p19q co‐deleted	40 (12.8)	3 (7.9)	0.679
Diffuse astrocytoma, IDH‐mutant	37 (11.9)	5 (13.2)	
Glioblastoma, IDH‐wildtype	235 (75.3)	30 (78.9)	
Molecular change in longitudinal data			
IDH mutation (first, second surgery)			
Mutation, mutation		8/38 (21.1)	
Wild‐type, wild‐type		30/38 (78.9)	
EGFR amplification	71/235 (30.2)		
EGFR amplification (first, second surgery)			
Amplification, amplification		6/30 (20)	
Non‐amplification, non‐amplification		16/30 (53.3)	
Non‐amplification, amplification		1/30 (3.3)	
Amplification, non‐amplification		7/30 (23.3)	

*Note*: Unless otherwise specified, the data are presented as numbers or numerators/denominators, with percentages in parentheses.

Abbreviations: EGFR, epidermal growth factor receptor; IDH, isocitrate dehydrogenase; SD, standard deviation.

* Statistical significance.

In the cross‐sectional set of 235 IDH‐wildtype patients, 71 (30.2%) showed EGFR amplification. Among the 30 IDH‐wildtype patients in the longitudinal set, EGFR amplification status remained unchanged between the first and second surgeries in 22 (73.3%) patients, whereas changes were reported in 8 (26.6%) patients. Imaging parameters related to IDH mutation status and EGFR amplification status in the cross‐sectional and longitudinal sets are summarized in Tables S1 and S2.

### 3.2 MRI Features Depicting Spatial Heterogeneity for IDH and EGFR Profiles in the Cross‐Sectional Cohort

Table 2 presents the intersection and Bhattacharyya distances between CET and NEL according to IDH mutation and EGFR amplification status. For IDH, a significantly larger intersection between CET and NEL was observed in the IDH mutant than in the IDH‐wildtype for nCBV (mutant vs. wild: 0.63 vs. 0.52), nCBF (0.68 vs. 0.60), and CMRO_2_ (0.67 vs. 0.58), indicating greater spatial heterogeneity in the IDH‐wildtype. In contrast, intersection values were significantly higher in the IDH‐wildtype for CTH (mutant vs. wild type: 0.51 vs. 0.60), MTT (0.45 vs. 0.53), and ADC (0.55 vs. 0.60) than in the IDH‐mutant type. The Bhattacharyya distances showed a similar pattern, with shorter distances observed in the IDH mutant for nCBV, nCBF, and CMRO_2_.

**TABLE 2:** Imaging features for spatial heterogeneity in IDH mutation and EGFR amplification status.

	IDH mutation status			EGFR amplification status in IDH‐wildtype		
	Mutation (*n* = 92)	Wild‐type (*n* = 296)	*p*	Amplification (*n* = 90)	Non‐amplification (*n* = 206)	*p*
Intersection						
nCBV	0.63 ± 0.21	0.52 ± 0.18	< 0.001*	0.50 ± 0.18	0.53 ± 0.18	0.169
nCBF	0.68 ± 0.19	0.60 ± 0.19	< 0.001*	0.58 ± 0.20	0.60 ± 0.19	0.457
CMRO_2_	0.67 ± 0.19	0.58 ± 0.18	< 0.001*	0.55 ± 0.18	0.59 ± 0.18	0.094
COV	0.57 ± 0.22	0.57 ± 0.20	0.927	0.55 ± 0.20	0.58 ± 0.20	0.229
CTH	0.51 ± 0.24	0.60 ± 0.20	0.001*	0.59 ± 0.22	0.60 ± 0.19	0.702
MTT	0.45 ± 0.27	0.53 ± 0.26	0.018*	0.54 ± 0.25	0.52 ± 0.26	0.547
OEF	0.67 ± 0.19	0.67 ± 0.16	0.928	0.66 ± 0.19	0.67 ± 0.14	0.73
TTP	0.30 ± 0.24	0.33 ± 0.27	0.374	0.32 ± 0.27	0.33 ± 0.27	0.817
ADC	0.55 ± 0.19	0.60 ± 0.18	0.01*	0.61 ± 0.19	0.60 ± 0.18	0.568
Bhattacharrya distance						
nCBV	0.34 ± 0.20	0.43 ± 0.17	< 0.001*	0.45 ± 0.17	0.42 ± 0.17	0.136
nCBF	0.30 ± 0.17	0.37 ± 0.18	0.003*	0.37 ± 0.18	0.36 ± 0.18	0.574
CMRO_2_	0.31 ± 0.18	0.38 ± 0.17	< 0.001*	0.41 ± 0.17	0.37 ± 0.17	0.088
COV	0.41 ± 0.21	0.40 ± 0.19	0.657	0.41 ± 0.19	0.39 ± 0.18	0.285
CTH	0.45 ± 0.22	0.38 ± 0.19	0.003*	0.38 ± 0.21	0.37 ± 0.18	0.696
MTT	0.51 ± 0.25	0.44 ± 0.24	0.019*	0.42 ± 0.24	0.44 ± 0.24	0.499
OEF	0.32 ± 0.19	0.31 ± 0.15	0.64	0.32 ± 0.19	0.31 ± 0.14	0.64
TTP	0.69 ± 0.23	0.66 ± 0.26	0.243	0.67 ± 0.26	0.65 ± 0.26	0.699
ADC	0.43 ± 0.17	0.38 ± 0.17	0.02*	0.38 ± 0.18	0.38 ± 0.16	0.764

*Note*: Data are presented as mean ± standard deviation.

Abbreviations: ADC, apparent diffusion coefficient; CMRO_2_, cerebral metabolic rate of oxygen; COV, coefficient of variation; CTH, capillary transit time heterogeneity; EGFR, epidermal growth factor receptor; IDH, isocitrate dehydrogenase; MTT, mean transit time; nCBF, normalized cerebral blood flow; nCBV, normalized cerebral blood volume; OEF, oxygen extraction fraction; TTP, time to peak.

* Statistical significance.

**FIGURE 2:**
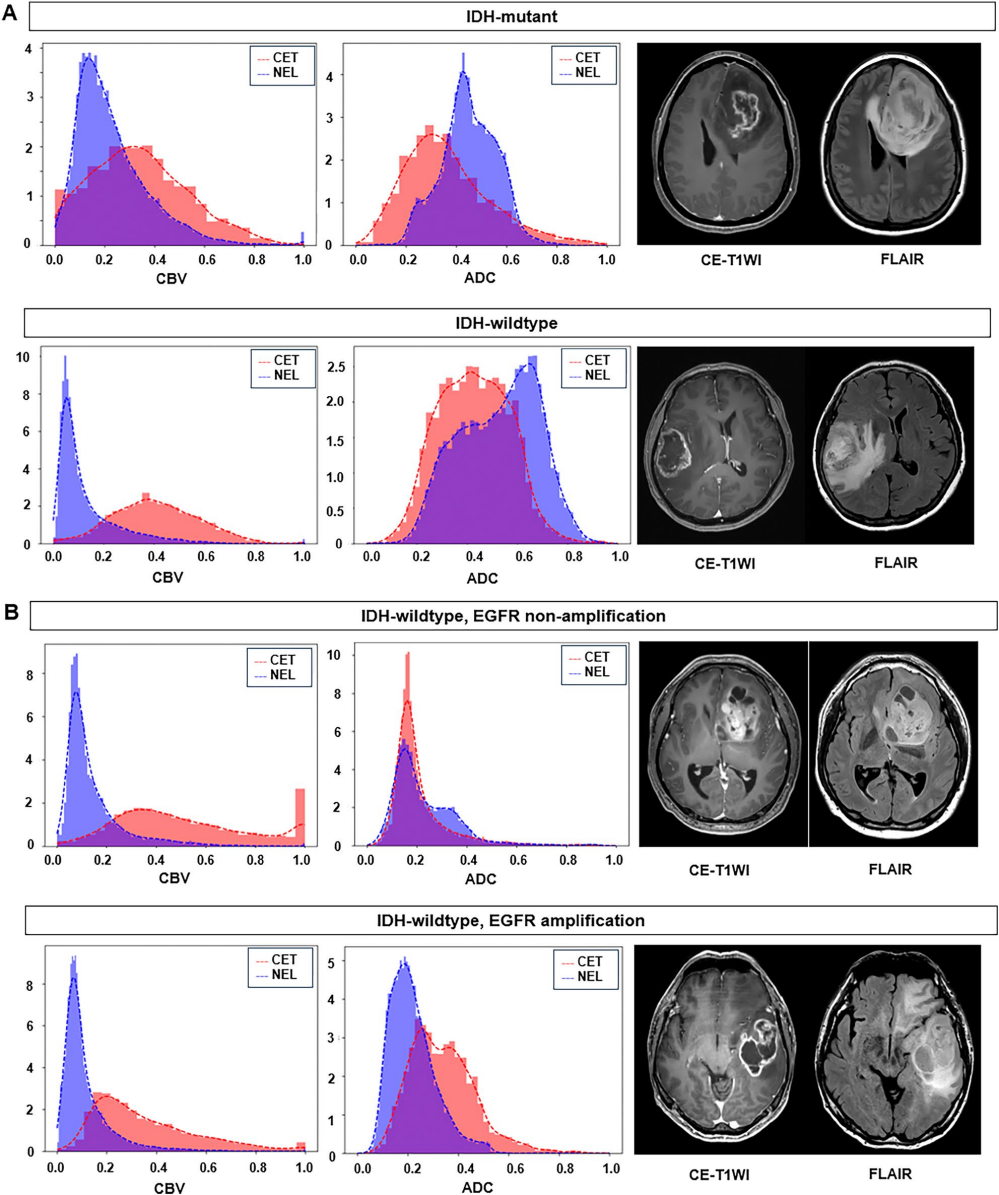
Imaging features reflecting spatial heterogeneity between contrast‐enhancing tumor (CET) and non‐enhancing lesion (NEL) according to IDH mutation status and EGFR amplification status using histogram analysis. (A) (Left) Comparison of the intersection between IDH‐mutant and IDH‐wildtype gliomas. Notably, IDH‐mutant gliomas show a larger intersection of CBV (IDH‐mutant vs. IDH‐wildtype: 0.66 vs. 0.31), whereas the IDH‐wildtype gliomas exhibit a larger intersection of ADC (IDH‐mutant vs. IDH‐wildtype: 0.59 vs. 0.72). (Right) On contrast‐enhanced T1‐weighted gradient‐echo imaging (CE‐T1WI) and FLAIR, both IDH‐mutant and IDH‐wild type tumors exhibit a necrotic enhancing mass with extensive FLAIR high signal intensity change. (B) (Left) Comparison of the intersections between EGFR‐amplified and non‐amplified gliomas, both IDH‐wildtype. The intersection of the CBV and ADC was similar between the two groups. The CBV map indicates no large overlap between CET and NEL, with no significant difference between the EGFR‐amplified and non‐amplified groups. In contrast, a large overlap was observed between CET and NEL on the ADC map, with no significant difference between the EGFR‐amplified and non‐amplified groups. (Right) On CE‐T1WI and FLAIR, both EGFR non‐amplified and amplified tumors exhibit a necrotic enhancing mass with extensive FLAIR high signal intensity infiltrative tumor. ADC, apparent diffusion coefficient; CBV, cerebral blood volume; EGFR, epidermal growth factor receptor; IDH, isocitrate dehydrogenase.

For EGFR amplification status in IDH‐wildtype tumors, no significant difference in spatial heterogeneity was observed between the ADC and perfusion parameters (Table 2). Figure 2 illustrates the spatiotemporal heterogeneity between CET and NEL for different IDH mutation and EGFR amplification statuses. IDH mutations exhibited a large overlap between CET and NEL on nCBV, whereas IDH‐wildtype exhibited a large overlap between regions on ADC. The EGFR amplification status showed no difference in overlap between the two regions on both the nCBV and ADC maps.

### 3.3 Temporally Stable MRI Features Reflecting Unchanged IDH and EGFR Profiles in the Longitudinal Cohort

All patients in the longitudinal set maintained consistent IDH mutation status between the first and second surgeries. A comparison of the initial and recurrence MRIs identified 251 stable features, with an ICC > 0.5. Detailed information on the ICC of these features is provided in the Supporting Information S2.

Among the 30 IDH‐wildtype patients in the longitudinal set, six exhibited consistent EGFR amplification across both the first and second surgeries, and 16 remained consistently EGFR non‐amplified in both surgeries. Consequently, 22 patients demonstrated a consistent EGFR status (either amplified or non‐amplified) at both time points. In these patients, 295 temporally stable features with an ICC of > 0.5 were identified.

### 3.4 Feature Selection for the Concordant Model and Best Model in the Cross‐Sectional Data

Of the 251 temporally stable features associated with IDH mutation status, 49 were selected using LASSO to construct a concordant model for IDH mutation categorization. Similarly, among the 295 temporally stable features associated with EGFR amplification status in IDH‐wildtype tumors, seven were selected using LASSO to build a concordant model for EGFR amplification categorization.

The LASSO was applied to a cross‐sectional set for feature selection. Thirty features were selected to build the best model for diagnosing IDH mutations, and 19 features were selected to construct the best model for diagnosing EGFR amplification status in IDH‐wildtype tumors. Details of the selected features are provided in Table S3.

### 3.5 Diagnostic Performance of the Concordant Model and Best Model

**FIGURE 3:**
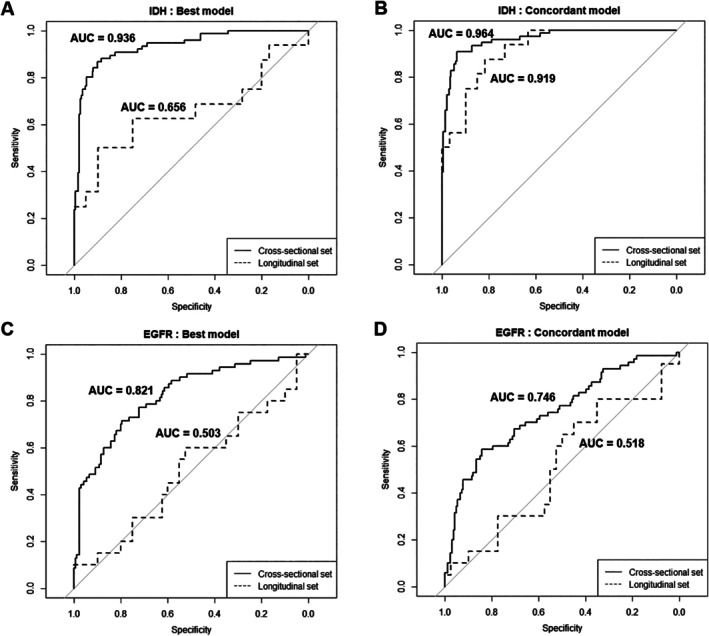
Diagnostic performance of the best and concordant models in the cross‐sectional and longitudinal sets for IDH mutation categorization (A, B) and EGFR amplification (C, D). EGFR, epidermal growth factor receptor; IDH, isocitrate dehydrogenase.

Table 3 and Figure 3 summarize the diagnostic performance of the concordant and best models in the cross‐sectional and longitudinal sets. In the cross‐sectional set, both models demonstrated high diagnostic performance for diagnosing IDH mutation status. The concordant model achieved an AUC of 0.964 (95% CI: 0.943–0.986), sensitivity of 90.8%, specificity of 93.6%, and accuracy of 93%. The best model exhibited an AUC of 0.936 (95% CI: 0.903–0.969), sensitivity of 86.8%, specificity of 90.3%, and accuracy of 89.4%. For diagnosing EGFR amplification in IDH‐wildtype tumors, the concordant model achieved an AUC of 0.746 (95% CI: 0.675–0.817), sensitivity of 58.6%, specificity of 84.3%, and accuracy of 76.7%. The best model had an AUC of 0.821 (95% CI: 0.761–0.881), sensitivity of 71.4%, specificity of 79.5%, and accuracy of 76.7%.

**TABLE 3:** Diagnostic performance of the concordant model and best model.

	Cross‐sectional set					Longitudinal set			
	AUC	Cutoff	Sensitivity	Specificity	Accuracy	AUC	Sensitivity	Specificity	Accuracy
	(95% CI)					(95% CI)			
IDH‐mutation									
Concordant model	0.964	0.326	90.8%	93.6%	93%	0.919	75%	86.7%	84.2%
	(0.943–0.986)		(69/76)	(221/236)	(290/312)	(0.853–0.985)	(12/16)	(52/60)	(64/76)
Best model	0.936	0.283	86.8%	90.3%	89.4%	0.656	50%	86.7%	79%
	(0.903–0.969)		(65/76)	(187/236)	(252/312)	(0.470–0.841)	(8/16)	(52/60)	(60/76)
EGFR amplification in IDH‐wildtype									
Concordant model	0.746	0.329	58.6%	84.3%	76.7%	0.518	30%	72.5%	58.3%
	(0.675–0.817)		(41/70)	(140/166)	(181/236)	(0.357–0.678)	(6/20)	(29/40)	(35/60)
Best model	0.821	0.359	71.4%	79.5%	77.1%	0.503	40%	62.5%	55%
	(0.761–0.881)		(50/70)	(132/166)	(182/236)	(0.34–0.665)	(8/20)	(25/40)	(33/60)

*Note*: Data in parentheses are 95% confidence intervals or numerator/denominator.

Abbreviations: AUC, area under the receiver operating characteristic curve; CI, confidence interval; EGFR, epidermal growth factor receptor; IDH, isocitrate dehydrogenase.

In the longitudinal set, the concordant model achieved an AUC of 0.919 (95% CI: 0.853–0.985), sensitivity of 75%, specificity of 86.7%, and accuracy of 84.2% in diagnosing IDH mutations. The best model demonstrated an AUC of 0.656 (95% CI: 0.47–0.841), sensitivity of 50%, specificity of 86.7%, and accuracy of 79%. Both models showed limited capabilities for diagnosing EGFR amplification in IDH‐wildtype tumors. The concordant model achieved an AUC of 0.518 (95% CI: 0.357–0.678), with a sensitivity of 30%, a specificity of 72.5%, and an accuracy of 58.3%. The best model had a similarly low AUC of 0.503 (95% CI: 0.34–0.665), sensitivity of 40%, specificity of 62.5%, and accuracy of 55%.

## 4 Discussion

This study evaluated spatial and temporal heterogeneity of diffusion and perfusion MRI parameters in CET and NEL to diagnose IDH mutation and EGFR amplification in adult‐type diffuse gliomas. The best model used MRI at a single time point (cross‐sectional data) for newly diagnosed gliomas, whereas the concordant model used MRI at two time points (longitudinal data) for both primary and recurrent tumors. For IDH mutations, both models demonstrated high performances in a cross‐sectional dataset of newly diagnosed gliomas. However, only the concordant model maintained a strong performance in the longitudinal dataset of recurrent gliomas, whereas the best model showed decreased performance. For EGFR amplification in IDH‐wildtype tumors, both models showed moderate performance in the cross‐sectional dataset but poor diagnostic performance in the longitudinal dataset. These results show that imaging features with temporal stability have high diagnostic performance for stable IDH mutations but poor performance for dynamic EGFR amplification, which changes over time.

Previous studies have similarly reported a better diagnostic performance for IDH mutations than for EGFR amplification using radiomics models. A radiomics model incorporating diffusion and conventional MRI achieved an accuracy of 0.76 for diagnosing IDH mutation, whereas accuracy was lower at 0.66 for EGFR amplification [32]. Likewise, a model utilizing both perfusion and diffusion MRI demonstrated a higher accuracy (0.92) for diagnosing IDH mutations but a lower accuracy (0.72) for EGFR amplification [33]. This discrepancy in performance between IDH mutations and EGFR amplification may be attributable to the greater spatial heterogeneity of EGFR amplification compared to the stable distribution of IDH mutations, making EGFR amplification inherently more challenging to diagnose. The current study offers insights into the molecular assessment of recurrent gliomas. For IDH mutations, a relatively stable temporal profile allows for more consistent diagnostic ability across primary and recurrent tumors, highlighting the importance of incorporating temporally stable imaging features into diagnostic models. In contrast, EGFR amplification is characterized by greater temporal heterogeneity, making diagnosis inherently challenging, particularly in recurrent gliomas. Although spatial heterogeneity analysis remains useful for diagnosing EGFR amplification in newly diagnosed tumors, addressing its temporal variability will require further refinement of imaging‐based diagnostic strategies.

In the current study, both perfusion and diffusion parameters were helpful in diagnosing IDH mutations in newly diagnosed gliomas. IDH‐mutant tumors exhibited lower nCBV in CET and higher ADC in both CET and NEL, consistent with previous studies [12, 34, 35]. Additionally, parameters related to microvasculature and hypoxia showed decreased CMRO_2_ in CET of IDH‐mutant tumors than in wild‐type tumors. This finding is consistent with that of a previous study indicating that high‐grade gliomas, which are more hypoxic, show increased CMRO_2_ and neovascularization, whereas IDH mutations in low‐grade gliomas are associated with decreased CMRO_2_ [36]. IDH mutations are early causative events in glioma formation, ubiquitously expressed throughout the tumor, and frequently retained during disease progression [37]. This relative temporal homogeneity likely explains the consistent diagnostic performance of the concordant model in diagnosing IDH mutation status in the longitudinal analysis. The perfusion and diffusion parameters observed in the current study were maintained in recurrent tumors, further demonstrating the stability of IDH‐mutated cells, even in recurrent settings.

In the current study, EGFR amplification was associated with high perfusion and low diffusion parameters, as evidenced by high nCBV, nCBF, and CMRO_2_, coupled with low ADC in EGFR‐amplified tumors. Notably, the best model showed higher nCBV and nCBF in NEL, whereas the concordant model showed higher nCBF and CMRO_2_ in NEL. These findings are consistent with those of a previous deep learning study that used complex time‐signal intensity curves from DSC imaging of immediate and distant peritumoral regions [15]. EGFRvIII‐positive tumors showed more homogeneity in time‐signal intensity curve‐based features between the immediate and distant peritumoral regions, whereas EGFRvIII‐negative tumors displayed greater heterogeneity. However, diagnosing EGFR amplification longitudinally remains challenging, even with models using temporally stable features selected from both initial and recurrent tumors. Two hypotheses may explain the temporal heterogeneity of EGFR amplification [7]. First, environmental pressures within the tumor microenvironment may be regional, leading to the focal emergence of EGFRvIII and other EGFR mutations promoting angiogenesis through oncogenic signaling. These selective pressures may favor specific EGFR mutations, such as EGFRvIII, in EGFR‐amplified tumor cells at later stages of tumor development. The second possibility is “mutational switching,” where one EGFR mutation replaces another upon tumor recurrence [38]. This process, recently described in glioblastoma, suggests the frequent occurrence and disappearance of EGFR mutations, contributing to temporal heterogeneity. The spatial heterogeneity in CET and peritumoral NEL, combined with temporal heterogeneity of EGFR amplification, complicates the use of physiologic MRI from both regions for accurately diagnosing EGFR amplification.

To further explore spatial heterogeneity, the intersection and Bhattacharyya distances between CET and NEL were calculated. IDH‐mutant tumors showed higher intersections in nCBV, nCBF, and CMRO_2_ between CET and NEL compared with IDH‐wildtype tumors. This increased overlap aligns with the ubiquitous expression of IDH mutations across the tumor [1]. Similar findings were reported in a previous study, where histogram analysis of rCBV‐voxels showed IDH‐mutant tumors clustering at lower values, whereas wild‐type tumors had a broader distribution [12]. Mutations in cancer‐associated IDH acquire neoactivity, producing 2‐hydroxyglutarate and leading to decreased activation of hypoxia‐inducible factor 1‐alpha [39], explaining the similarly high CMRO_2_ in both CET and NEL. In the current study, the overlap of ADC values between CET and NEL regions was greater in IDH‐wildtype than in IDH‐mutant tumors. This observation is consistent with the presence of substantial tumor infiltration in FLAIR hyperintensity regions of IDH‐wildtype glioblastomas, contributing to their aggressive biological behavior [40, 41]. Diffuse tumor infiltration and microvascular proliferation in the NEL of IDH‐wildtype gliomas likely resulted in comparable ADC values as those observed in the CET, explaining the greater overlap. Regarding EGFR amplification, no significant difference was observed between non‐amplified and amplified tumors, which could be attributed to the sporadic expression of EGFR amplification [7], unlike the ubiquitous IDH mutations, making detection of differences between CET and NEL regions challenging.

## 5 Limitations

First, the small sample size and absence of an external validation set, particularly in the longitudinal analysis, limit the generalizability of the findings. Publicly available longitudinal datasets with pathological validation are scarce; therefore, every effort was made to include 38 patients with longitudinal pathological data from primary and recurrent tumors. Nonetheless, a multicenter study with a larger patient cohort for longitudinal datasets is warranted. Second, the prognostic relevance of the selected imaging features was not directly assessed for mid‐ to long‐term clinical outcomes. Future studies with longitudinal clinical follow‐up data are needed to validate the prognostic implications of our findings. Third, microvascular and oxygenation parameters are not widely available, and combining them with dynamic contrast enhancement parameters may improve utility. Finally, temporal heterogeneity, especially for EGFR, should be validated through biopsies from multiple locations on the specimen and correlated with EGFR amplification levels.

## 6 Conclusion

This study highlights the challenges in diagnosing EGFR amplification status in recurrent gliomas. Although IDH mutation status in newly diagnosed and recurrent gliomas can be reliably assessed using multi‐parametric MRI, the greater spatial and temporal variability of EGFR amplification complicates its imaging‐based characterization.

## Supporting information


Data S1.

Data S2.

## References

[1] D. N. Louis , A. Perry , P. Wesseling , et al., “The 2021 WHO Classification of Tumors of the Central Nervous System: A Summary,” Neuro‐Oncology 23 (2021): 1231–1251. 34185076 10.1093/neuonc/noab106 PMC8328013

[2] W. Wick , P. Roth , C. Hartmann , et al., “Long‐Term Analysis of the NOA‐04 Randomized Phase III Trial of Sequential Radiochemotherapy of Anaplastic Glioma With PCV or Temozolomide,” Neuro‐Oncology 18 (2016): 1529–1537. 27370396 10.1093/neuonc/now133 PMC5063521

[3] Z. An , O. Aksoy , T. Zheng , Q. W. Fan , and W. A. Weiss , “Epidermal Growth Factor Receptor and EGFRvIII in Glioblastoma: Signaling Pathways and Targeted Therapies,” Oncogene 37 (2018): 1561–1575. 29321659 10.1038/s41388-017-0045-7 PMC5860944

[4] J. Li , R. Liang , C. Song , Y. Xiang , and Y. Liu , “Prognostic Significance of Epidermal Growth Factor Receptor Expression in Glioma Patients,” Oncotargets and Therapy 11 (2018): 731–742. 29445288 10.2147/OTT.S155160 PMC5808691

[5] C. N. Kersch , M. Kim , J. Stoller , R. F. Barajas, Jr. , and J. E. Park , “Imaging Genomics of Glioma Revisited: Analytic Methods to Understand Spatial and Temporal Heterogeneity,” American Journal of Neuroradiology 45 (2024): 537–548. 38548303 10.3174/ajnr.A8148 PMC11288537

[6] A. L. Cohen , S. L. Holmen , and H. Colman , “IDH1 and IDH2 Mutations in Gliomas,” Current Neurology and Neuroscience Reports 13 (2013): 345. 23532369 10.1007/s11910-013-0345-4 PMC4109985

[7] E. Eskilsson , G. V. Røsland , G. Solecki , et al., “EGFR Heterogeneity and Implications for Therapeutic Intervention in Glioblastoma,” Neuro‐Oncology 20 (2018): 743–752. 29040782 10.1093/neuonc/nox191 PMC5961011

[8] M. J. van den Bent , Y. Gao , M. Kerkhof , et al., “Changes in the EGFR Amplification and EGFRvIII Expression Between Paired Primary and Recurrent Glioblastomas,” Neuro‐Oncology 17 (2015): 935–941. 25691693 10.1093/neuonc/nov013 PMC5762005

[9] P. S. LaViolette , N. J. Mickevicius , E. J. Cochran , et al., “Precise Ex Vivo Histological Validation of Heightened Cellularity and Diffusion‐Restricted Necrosis in Regions of Dark Apparent Diffusion Coefficient in 7 Cases of High‐Grade Glioma,” Neuro‐Oncology 16 (2014): 1599–1606. 25059209 10.1093/neuonc/nou142 PMC4232087

[10] X. Ma , K. Cheng , G. Cheng , et al., “Apparent Diffusion Coefficient as Imaging Biomarker for Identifying IDH Mutation, 1p19q Codeletion, and MGMT Promoter Methylation Status in Patients With Glioma,” Journal of Magnetic Resonance Imaging 58 (2023): 732–738. 36594577 10.1002/jmri.28589

[11] Y. W. Park , J. E. Park , S. S. Ahn , et al., “Magnetic Resonance Imaging Parameters for Noninvasive Prediction of Epidermal Growth Factor Receptor Amplification in Isocitrate Dehydrogenase‐Wild‐Type Lower‐Grade Gliomas: A Multicenter Study,” Neurosurgery 89 (2021): 257–265. 33913501 10.1093/neuros/nyab136

[12] P. Kickingereder , F. Sahm , A. Radbruch , et al., “IDH Mutation Status Is Associated With a Distinct Hypoxia/Angiogenesis Transcriptome Signature Which Is Non‐Invasively Predictable With rCBV Imaging in Human Glioma,” Scientific Reports 5 (2015): 16238. 26538165 10.1038/srep16238 PMC4633672

[13] A. Gupta , R. J. Young , A. D. Shah , et al., “Pretreatment Dynamic Susceptibility Contrast MRI Perfusion in Glioblastoma: Prediction of EGFR Gene Amplification,” Clinical Neuroradiology 25 (2015): 143–150. 10.1007/s00062-014-0289-3 PMC4712066 24474262

[14] T. C. Oughourlian , J. Yao , A. Hagiwara , et al., “Relative Oxygen Extraction Fraction (rOEF) MR Imaging Reveals Higher Hypoxia in Human Epidermal Growth Factor Receptor (EGFR) Amplified Compared With Non‐Amplified Gliomas,” Neuroradiology 63 (2021): 857–868. 33106922 10.1007/s00234-020-02585-8 PMC8071834

[15] S. Bakas , H. Akbari , J. Pisapia , et al., “In Vivo Detection of EGFRvIII in Glioblastoma via Perfusion Magnetic Resonance Imaging Signature Consistent With Deep Peritumoral Infiltration: The φ‐Index,” Clinical Cancer Research 23 (2017): 4724–4734. 28428190 10.1158/1078-0432.CCR-16-1871 PMC5559313

[16] J. E. Villanueva‐Meyer , M. D. Wood , B. S. Choi , et al., “MRI Features and IDH Mutational Status of Grade II Diffuse Gliomas: Impact on Diagnosis and Prognosis,” AJR. American Journal of Roentgenology 210 (2018): 621–628. 29261348 10.2214/AJR.17.18457 PMC5823758

[17] Z. Xing , X. Yang , D. She , Y. Lin , Y. Zhang , and D. Cao , “Noninvasive Assessment of IDH Mutational Status in World Health Organization Grade II and III Astrocytomas Using DWI and DSC‐PWI Combined With Conventional MR Imaging,” AJNR. American Journal of Neuroradiology 38 (2017): 1138–1144. 28450436 10.3174/ajnr.A5171 PMC7960080

[18] A. S. Haider , M. van den Bent , P. Y. Wen , et al., “Toward a Standard Pathological and Molecular Characterization of Recurrent Glioma in Adults: A Response Assessment in Neuro‐Oncology Effort,” Neuro‐Oncology 22 (2020): 450–456. 31844891 10.1093/neuonc/noz233 PMC7158649

[19] F. Isensee , M. Schell , I. Pflueger , et al., “Automated Brain Extraction of Multisequence MRI Using Artificial Neural Networks,” Human Brain Mapping 40 (2019): 4952–4964. 31403237 10.1002/hbm.24750 PMC6865732

[20] U. Baid , S. Ghodasara , S. Mohan , et al., “The RSNA‐ASNR‐MICCAI BraTS 2021 Benchmark on Brain Tumor Segmentation and Radiogenomic Classification,” (2021), In Press.

[21] F. Isensee , P. F. Jaeger , S. A. A. Kohl , J. Petersen , and K. H. Maier‐Hein , “ nnU‐Net: A Self‐Configuring Method for Deep Learning‐Based Biomedical Image Segmentation,” Nature Methods 18 (2021): 203–211. 33288961 10.1038/s41592-020-01008-z

[22] P. Kickingereder , F. Isensee , I. Tursunova , et al., “Automated Quantitative Tumour Response Assessment of MRI in Neuro‐Oncology With Artificial Neural Networks: A Multicentre, Retrospective Study,” Lancet Oncology 20 (2019): 728–740. 30952559 10.1016/S1470-2045(19)30098-1

[23] D. Bonekamp , K. Mouridsen , A. Radbruch , et al., “Assessment of Tumor Oxygenation and Its Impact on Treatment Response in Bevacizumab‐Treated Recurrent Glioblastoma,” Journal of Cerebral Blood Flow and Metabolism 37 (2017): 485–494. 26861817 10.1177/0271678X16630322 PMC5381446

[24] A. Stadlbauer , K. Mouridsen , A. Doerfler , et al., “Recurrence of Glioblastoma Is Associated With Elevated Microvascular Transit Time Heterogeneity and Increased Hypoxia,” Journal of Cerebral Blood Flow and Metabolism: Official Journal of the International Society of Cerebral Blood Flow and Metabolism 38 (2018): 422–432. 28273720 10.1177/0271678X17694905 PMC5851132

[25] C. J. Galban , T. L. Chenevert , C. R. Meyer , et al., “The Parametric Response Map Is an Imaging Biomarker for Early Cancer Treatment Outcome,” Nature Medicine 15 (2009): 572–576. 10.1038/nm.1919 PMC3307223 19377487

[26] J. J. M. van Griethuysen , A. Fedorov , C. Parmar , et al., “Computational Radiomics System to Decode the Radiographic Phenotype,” Cancer Research 77 (2017): e104–e107. 29092951 10.1158/0008-5472.CAN-17-0339 PMC5672828

[27] A. Bhattacharyya , “On a Measure of Divergence Between Two Multinomial Populations,” Sankhyā: The Indian Journal of Statistics 7 (1946): 401–406.

[28] F. B. Furnari , T. Fenton , R. M. Bachoo , et al., “Malignant Astrocytic Glioma: Genetics, Biology, and Paths to Treatment,” Genes and Development 21 (2007): 2683–2710. 17974913 10.1101/gad.1596707

[29] J. Eng , “Sample Size Estimation: How Many Individuals Should Be Studied?,” Radiology 227 (2003): 309–313. 12732691 10.1148/radiol.2272012051

[30] C. Xue , J. Yuan , G. G. Lo , et al., “Radiomics Feature Reliability Assessed by Intraclass Correlation Coefficient: A Systematic Review,” Quantitative Imaging in Medicine and Surgery 11 (2021): 4431–4460. 34603997 10.21037/qims-21-86 PMC8408801

[31] J. E. Park , S. Y. Park , H. J. Kim , and H. S. Kim , “Reproducibility and Generalizability in Radiomics Modeling: Possible Strategies in Radiologic and Statistical Perspectives,” Korean Journal of Radiology 20 (2019): 1124–1137. 31270976 10.3348/kjr.2018.0070 PMC6609433

[32] S. Kihira , N. M. Tsankova , A. Bauer , et al., “Multiparametric MRI Texture Analysis in Prediction of Glioma Biomarker Status: Added Value of MR Diffusion,” Neuro‐Oncology Advances 3 (2021): vdab051. 34056604 10.1093/noajnl/vdab051 PMC8156980

[33] E. Calabrese , J. D. Rudie , A. M. Rauschecker , et al., “Combining Radiomics and Deep Convolutional Neural Network Features From Preoperative MRI for Predicting Clinically Relevant Genetic Biomarkers in Glioblastoma,” Neuro‐Oncology Advances 4 (2022): vdac060. 35611269 10.1093/noajnl/vdac060 PMC9122791

[34] D. A. Gutman , L. A. Cooper , S. N. Hwang , et al., “MR Imaging Predictors of Molecular Profile and Survival: Multi‐Institutional Study of the TCGA Glioblastoma Data Set,” Radiology 267 (2013): 560–569. 23392431 10.1148/radiol.13120118 PMC3632807

[35] P. Kickingereder , D. Bonekamp , M. Nowosielski , et al., “Radiogenomics of Glioblastoma: Machine Learning‐Based Classification of Molecular Characteristics by Using Multiparametric and Multiregional MR Imaging Features,” Radiology 281, no. 3 (2016): 907–918, 10.1148/radiol.2016161382. 27636026

[36] A. Stadlbauer , M. Zimmermann , M. Kitzwögerer , et al., “MR Imaging‐Derived Oxygen Metabolism and Neovascularization Characterization for Grading and IDH Gene Mutation Detection of Gliomas,” Radiology 283 (2017): 799–809. 27982759 10.1148/radiol.2016161422

[37] J. J. Miller , L. N. Gonzalez Castro , S. McBrayer , et al., “Isocitrate Dehydrogenase (IDH) Mutant Gliomas: A Society for Neuro‐Oncology (SNO) Consensus Review on Diagnosis, Management, and Future Directions,” Neuro‐Oncology 25, no. 1 (2023): 4–25, 10.1093/neuonc/noac207. 36239925 PMC9825337

[38] J. Wang , E. Cazzato , E. Ladewig , et al., “Clonal Evolution of Glioblastoma Under Therapy,” Nature Genetics 48 (2016): 768–776. 27270107 10.1038/ng.3590 PMC5627776

[39] S. Zhao , Y. Lin , W. Xu , et al., “Glioma‐Derived Mutations in IDH1 Dominantly Inhibit IDH1 Catalytic Activity and Induce HIF‐1alpha,” Science 324 (2009): 261–265. 19359588 10.1126/science.1170944 PMC3251015

[40] G. Broggi , R. Altieri , V. Barresi , et al., “Histologic Definition of Enhancing Core and FLAIR Hyperintensity Region of Glioblastoma, IDH‐Wild Type: A Clinico‐Pathologic Study on a Single‐Institution Series,” Brain Sciences 13, no. 2 (2023): 248, 10.3390/brainsci13020248. 36831791 PMC9954517

[41] R. Altieri , G. Broggi , F. Certo , et al., “Anatomical Distribution of Cancer Stem Cells Between Enhancing Nodule and FLAIR Hyperintensity in Supratentorial Glioblastoma: Time to Recalibrate the Surgical Target?,” Neurosurgical Review 45 (2022): 3709–3716. 36171505 10.1007/s10143-022-01863-8

