# Supplementary material for: Imaging‐Based Molecular Characterization of Adult‐Type Diffuse Glioma Using Diffusion and Perfusion MRI in Pre‐ and Post‐Treatment Stage Considering Spatial and Temporal Heterogeneity
Source: J Magn Reson Imaging. 2025 Apr 8;62(2):468–79. doi: 10.1002/jmri.29781 (PMC12276639; doi:10.1002/jmri.29781)
Supplement: Supplementary file 1 — Data S1. [file JMRI-62-468-s001.docx]

**Supplementary Materials**

**Supplementary Material 1. MRI Acquisition**

T2-weighted and FLAIR images were acquired using spin-echo sequences with the following parameters: T2-weighted: repetition time (TR)/echo time (TE), 3000/100 ms; field of view (FOV), 240 × 240 mm; matrix, 256 × 256; slice thickness, 4 mm without a gap; FLAIR: TR/TE 10000/130 ms, inversion time 2800 ms, FOV 240 × 240 mm; matrix, 256 × 256; and slice thickness, 4 mm without a gap.

High-resolution anatomical three-dimensional volume images were acquired using gradient-echo T1-weighted sequences with and without a gadolinium-based contrast agent with the following parameters: TR/TE 9.8/4.6 ms; flip angle, 10°; FOV, 256 × 256 mm; matrix, 512 × 512; and slice thickness, 1 mm with no gap.

Diffusion-weighted imaging was performed using the following parameters: TR/TE, 3000/56 ms; diffusion gradient encoding, b = 0 and 1000 s/mm^2^; FOV, 250 mm; slice thickness/gap, 5 mm/2 mm; matrix, 256 × 256; and acquisition time, 39 s.

Dynamic susceptibility contrast perfusion MRI was performed using a gradient-echo echo-planar sequence with the following parameters: TR/TE, 1500/40 ms; flip angle, 35°; FOV, 24 cm; slice thickness/gap, 5 mm/2 mm; and matrix, 128 × 128. The total acquisition time for DSC–MRI was 1 min 54 s.

**Supplementary Table 1. Comparison of Imaging Parameters According to IDH Mutation Status**

|  | **Cross-sectional set (n = 312)** | |  | **Longitudinal set (n = 76)** | |  |  |
| --- | --- | --- | --- | --- | --- | --- | --- |
|  | **IDH mutation status** | |  | **IDH mutation status** | |  |  |
|  | **Mutation (n = 77)** | **Wild-type (n = 235)** | ***P* Value** | **Mutation (n = 16)** | **Wild-type (n = 60)** | ***P* Value** | ***P* Value** |
| **CET** | | | | | | | |
| nCBV | 2.16 ± 1.12 | 3.7 ± 1.9 | *<0.001** | 1.9 ± 0.9 | 2.5 ± 1.4 | *0.093* | *<0.001** |
| nCBF | 2.51 ± 1.93 | 4.1 ± 3.3 | *<0.001** | 2.2 ± 2.3 | 2.5 ± 1.7 | *0.605* | *0.001** |
| CMRO_2_ | 4322.8 ± 4429.3 | 7074.1 ± 6881.9 | *0.001** | 4069.7 ± 3416.8 | 6558.4 ± 5778.8 | *0.105* | *0.654* |
| COV | 1989.4 ± 2973.4 | 2418.9 ± 3453.5 | *0.328* | 892.7 ± 2452.4 | 1223.3 ± 2665 | *0.656* | *0.005** |
| CTH | 1317.1 ± 2678.9 | 1287.2 ± 1958.6 | *0.916* | 427.9 ± 1260.9 | 324.7 ± 1035.3 | *0.736* | *<0.001** |
| MTT | 815.9 ± 1102.3 | 799.9 ± 838.3 | *0.893* | 1236.2 ± 2217.4 | 854.7 ±  715 | *0.256* | *0.291* |
| OEF | 5552.2 ± 7703.3 | 6575.9 ± 7562.9 | *0.306* | 5351.7 ± 8742.4 | 3755.1 ± 6371 | *0.415* | *0.02** |
| TTP | 3134.43 ± 4849.8 | 3444.8 ± 4934.6 | *0.631* | 566.4 ± 2175.1 | 888 ± 2908.4 | *0.682* | *<0.001** |
| ADC | 1277.15 ± 242.4 | 1129.7 ± 224.3 | *<0.001** | 1361.6 ± 230 | 1263.8 ± 219.1 | *0.121* | *<0.001** |
| **NEL** | | | | | | | |
| nCBV | 1.86 ± 0.61 | 1.91 ± 0.9 | *0.637* | 1.6 ± 0.7 | 1.4 ± 0.6 | *0.132* | *<0.001** |
| nCBF | 2.51 ± 1.22 | 2.4 ± 1.7 | *0.466* | 2.3 ± 2.1 | 1.7 ± 1.1 | *0.111* | *0.002** |
| CMRO_2_ | 4266.6 ± 3921.8 | 4048.9 ± 3874.9 | *0.67* | 3986.8 ± 3391.7 | 4190.8 ± 3685 | *0.842* | *0.927* |
| COV | 2062.6 ± 3064.4 | 2166 ± 3023.1 | *0.795* | 821.7 ± 2242 | 1129.8 ± 2477.8 | *0.654* | *0.004** |
| CTH | 884.9 ± 1552 | 1000.3 ± 1451.4 | *0.552* | 230.4 ± 633.7 | 269.4 ± 835.9 | *0.863* | *<0.001** |
| MTT | 556.2 ± 567.4 | 616.7 ± 591.2 | *0.432* | 690.9 ± 565.5 | 702 ± 559.4 | *0.944* | *0.188* |
| OEF | 4917.8 ± 6469.7 | 6283 ± 7057.6 | *0.134* | 4101.9 ± 6423.3 | 3774.7 ± 6393.7 | *0.856* | *0.016** |
| TTP | 2993.7 ± 4612 | 3398.6 ± 4876.9 | *0.522* | 551 ± 2116.7 | 893.4 ± 2923.5 | *0.663* | *<0.001** |
| ADC | 1167.8 ± 127 | 1098.4 ± 153.9 | *<0.001** | 1262.5 ± 162.4 | 1212 ± 153.7 | *0.252* | *<0.001** |
| **Bhattacharrya distance** | | | | | | | |
| nCBV | 0.34 ± 0.2 | 0.44 ± 0.17 | *<0.001** | 0.36 ±0.21 | 0.39 ± 0.16 | *0.556* | *0.207* |
| nCBF | 0.29 ± 0.17 | 0.37 ± 0.18 | *<0.001** | 0.37 ±0.2 | 0.35 ±0.19 | *0.679* | *0.894* |
| CMRO_2_ | 0.31 ± 0.18 | 0.39 ± 0.17 | *<0.001** | 0.32 ±0.18 | 0.36 ±0.17 | *0.484* | *0.419* |
| COV | 0.41 ± 0.21 | 0.4 ±0.18 | *0.87* | 0.41 ±0.21 | 0.37 ±0.19 | *0.474* | *0.325* |
| CTH | 0.46 ± 0.22 | 0.38 ± 0.19 | *0.002** | 0.41 ±0.22 | 0.37 ±0.17 | *0.406* | *0.461* |
| MTT | 0.52 ± 0.25 | 0.44 ±0.24 | *0.017** | 0.44 ±0.25 | 0.41 ±0.25 | *0.683* | *0.133* |
| OEF | 0.33 ± 0.18 | 0.32 ±0.16 | *0.802* | 0.3 ±0.21 | 0.27 ±0.14 | *0.626* | *0.034** |
| TTP | 0.68 ±0.23 | 0.66 ±0.25 | *0.573* | 0.76 ±0.21 | 0.63 ±0.29 | *0.104* | *0.864* |
| ADC | 0.44 ±0.17 | 0.39 ±0.17 | *0.024** | 0.37 ±0.18 | 0.34 ±0.15 | *0.467* | *0.012** |
| **Intersection** | | | | | | | |
| nCBV | 0.64 ± 0.21 | 0.51 ± 0.18 | *<0.001** | 0.62 ±0.23 | 0.56 ±0.18 | *0.278* | *0.231* |
| nCBF | 0.69 ± 0.18 | 0.59 ± 0.19 | *<0.001** | 0.6 ±0.22 | 0.61 ± 0.2 | *0.769* | *0.81* |
| CMRO_2_ | 0.67 ± 0.19 | 0.57 ± 0.18 | *<0.001** | 0.66 ±0.2 | 0.6 ±0.19 | *0.289* | *0.481* |
| COV | 0.57 ± 0.22 | 0.56 ± 0.2 | *0.797* | 0.57 ±0.23 | 0.61 ±0.21 | *0.472* | *0.193* |
| CTH | 0.5 ±0.24 | 0.59 ± 0.2 | *0.001** | 0.56 ±0.23 | 0.6 ±0.19 | *0.446* | *0.338* |
| MTT | 0.43 ± 0.27 | 0.52 ±0.26 | *0.013** | 0.54 ±0.26 | 0.56 ±0.27 | *0.813* | *0.077* |
| OEF | 0.66 ± 0.19 | 0.66 ± 0.16 | *0.827* | 0.7 ±0.2 | 0.71 ±0.14 | *0.699* | *0.012** |
| TTP | 0.31 ±0.24 | 0.32 ±0.26 | *0.799* | 0.22 ±0.21 | 0.35 ±0.3 | *0.114* | *0.82* |
| ADC | 0.53 ±0.18 | 0.59 ±0.19 | *0.009** | 0.62 ±0.19 | 0.65 ±0.15 | *0.508* | *0.077* |

Data are presented as mean ± standard deviation.

IDH, isocitrate dehydrogenase; EGFR, epidermal growth factor receptor; CET, contrast-enhancing tumor; NEL, nonenhancing lesion; nCBV, normalized cerebral blood volume; nCBF, normalized cerebral blood flow, CMRO_2_, cerebral metabolic rate of oxygen; COV, coefficient of variation; CTH, capillary transit time heterogeneity; MTT, mean transit time; OEF, oxygen extraction fraction; TTP, time to peak; ADC, apparent diffusion coefficient.

*indicates statistical significance.

**Supplementary Table 2. Comparison of Imaging Parameters According to EGFR Amplification Status**

|  | **Cross-sectional set (n = 235)** | |  | **Longitudinal set (n = 60)** | |  |  |
| --- | --- | --- | --- | --- | --- | --- | --- |
|  | **EGFR amplification status in IDH-wildtype** | |  | **EGFR amplification status in IDH-wildtype** | |  |  |
|  | **Amplification (n = 69)** | **Non-amplification (n = 166)** | ***P* Value** | **Amplification (n = 20)** | **Non-amplification (n = 40)** | ***P* Value** | ***P* Value** |
| **CET** |  |  |  |  |  |  |  |
| nCBV | 4.5 ± 2.3 | 3.3 ± 1.7 | *<0.001** | 2.7 ± 1.6 | 2.4 ± 1.3 | *0.384* | *<0.001** |
| nCBF | 5.1 ± 3.5 | 3.7 ±3.1 | *0.003** | 2.7 ± 2.3 | 2.4 ± 1.4 | *0.607* | *<0.001** |
| CMRO_2_ | 9757.7 ± 7523.2 | 5958.7 ± 6291 | *<0.001** | 6461.2 ± 6364.1 | 6607.1 ± 5548.1 | *0.927* | *0.594* |
| COV | 2504.8 ± 3513.6 | 2383.2 ± 3438.3 | *0.807* | 1168.1 ± 2903.9 | 1250.9 ± 2575.7 | *0.911* | *0.013** |
| CTH | 1449.8 ± 1963.7 | 1219.5 ± 1958.3 | *0.413* | 572.3 ± 1209.5 | 200.9 ±928.3 | *0.193* | *<0.001** |
| MTT | 879.1 ± 780.9 | 766.9 ± 861.2 | *0.351* | 853.3 ± 746.8 | 855.4 ± 708.3 | *0.992* | *0.642* |
| OEF | 6314.4 ± 7260.1 | 6684.6 ± 7703.8 | *0.733* | 5091.8 ± 7198.4 | 3086.8 ± 5898 | *0.254* | *0.008** |
| TTP | 3712.3 ± 5074.6 | 3333.6 ± 4886.5 | *0.593* | 1663.8 ± 4030.7 | 500.1 ± 2101.9 | *0.145* | *<0.001** |
| ADC | 1083.1 ± 201.5 | 1149 ± 231 | *0.04** | 1274.6 ± 214.2 | 1258.3 ± 224 | *0.789* | *<0.001** |
| **NEL** |  |  |  |  |  |  |  |
| nCBV | 2.2 ± 1.1 | 1.8 ±0.8 | *0.003** | 1.3 ± 0.7 | 1.4 ± 0.5 | *0.752* | *<0.001** |
| nCBF | 2.9 ± 2 | 2.1 ± 1.4 | *0.002** | 1.4 ±1.1 | 1.8 ± 1.1 | *0.203* | *0.002** |
| CMRO_2_ | 5276.8 ± 4184.1 | 3538.5 ± 3631.3 | *0.002** | 3708.6 ± 4209.3 | 4431.9 ± 3425.2 | *0.478* | *0.798* |
| COV | 2241.7 ± 3040 | 2134.6 ± 3024.7 | *0.805* | 1031.7 ± 2607 | 1178.8 ± 2443.3 | *0.831* | *0.015** |
| CTH | 1098.2 ± 1475.4 | 959.6 ± 1443.8 | *0.506* | 492.7 ± 1050.7 | 157.8 ± 693.4 | *0.145* | *<0.001** |
| MTT | 694.4 ± 627.6 | 584.5 ± 574.3 | *0.195* | 697.5 ±608.7 | 704.3 ± 541.1 | *0.965* | *0.314* |
| OEF | 5913.1 ± 6777.4 | 6436.7 ± 7185.2 | *0.606* | 5081 ± 7149.4 | 3121.6 ± 5969.1 | *0.267* | *0.013** |
| TTP | 3699.4 ± 5090.7 | 3273.5 ± 4795.5 | *0.543* | 1669.4 ± 4036.3 | 505.4 ± 2128.2 | *0.148* | *<0.001** |
| ADC | 1109 ± 151.2 | 1094 ± 155.2 | *0.498* | 1229.2 ± 140.1 | 1203.4 ± 161.1 | *0.545* | *<0.001** |
| **Bhattacharrya distance** | | | | | | | |
| nCBV | 0.47 ± 0.17 | 0.43 ± 0.17 | *0.071* | 0.39 ± 0.17 | 0.39 ± 0.16 | *0.998* | *0.052* |
| nCBF | 0.38 ± 0.18 | 0.37 ± 0.17 | *0.512* | 0.36 ± 0.18 | 0.35 ± 0.19 | *0.848* | *0.39* |
| CMRO_2_ | 0.42 ± 0.17 | 0.37 ± 0.16 | *0.054* | 0.37 ± 0.17 | 0.35 ± 0.18 | *0.784* | *0.197* |
| COV | 0.41 ± 0.2 | 0.4 ± 0.18 | *0.562* | 0.41 ± 0.18 | 0.35 ± 0.2 | *0.238* | *0.244* |
| CTH | 0.4 ± 0.22 | 0.37 ± 0.18 | *0.396* | 0.35 ± 0.19 | 0.38 ± 0.17 | *0.471* | *0.786* |
| MTT | 0.44 ± 0.25 | 0.44 ± 0.24 | *0.989* | 0.34 ± 0.2 | 0.44 ± 0.26 | *0.144* | *0.327* |
| OEF | 0.34 ± 0.2 | 0.32 ± 0.14 | *0.387* | 0.26 ± 0.14 | 0.28 ± 0.14 | *0.682* | *0.034** |
| TTP | 0.69 ± 0.25 | 0.65 ± 0.26 | *0.282* | 0.57 ± 0.28 | 0.66 ± 0.29 | *0.255* | *0.467* |
| ADC | 0.38 ± 0.19 | 0.4 ± 0.16 | *0.583* | 0.35 ± 0.15 | 0.34 ± 0.15 | *0.691* | *0.046** |
| **Intersection** | | | | | | | |
| nCBV | 0.48 ±0.17 | 0.53 ± 0.19 | *0.105* | 0.56 ± 0.19 | 0.56 ± 0.18 | *0.88* | *0.069* |
| nCBF | 0.57 ± 0.2 | 0.6 ± 0.19 | *0.446* | 0.6 ± 0.2 | 0.62 ± 0.21 | *0.692* | *0.405* |
| CMRO_2_ | 0.54 ± 0.18 | 0.58 ± 0.18 | *0.068* | 0.59 ±0.18 | 0.61 ± 0.2 | *0.671* | *0.251* |
| COV | 0.55 ± 0.21 | 0.57 ± 0.19 | *0.606* | 0.55 ± 0.19 | 0.64 ± 0.21 | *0.106* | *0.12* |
| CTH | 0.58 ± 0.23 | 0.6 ± 0.19 | *0.46* | 0.62 ± 0.21 | 0.6 ± 0.18 | *0.615* | *0.697* |
| MTT | 0.52 ± 0.26 | 0.52 ± 0.26 | *0.971* | 0.63 ± 0.21 | 0.53 ± 0.28 | *0.18* | *0.26* |
| OEF | 0.65 ± 0.2 | 0.66 ± 0.14 | *0.463* | 0.72 ±0.14 | 0.71 ± 0.15 | *0.744* | *0.011** |
| TTP | 0.3 ± 0.25 | 0.33 ± 0.27 | *0.403* | 0.41 ± 0.3 | 0.32 ± 0.3 | *0.316* | *0.41* |
| ADC | 0.61 ± 0.2 | 0.59 ± 0.18 | *0.431* | 0.64 ± 0.16 | 0.65 ± 0.15 | *0.833* | *0.038** |

Data are presented as mean ± standard deviation.

IDH, isocitrate dehydrogenase; EGFR, epidermal growth factor receptor; CET, contrast-enhancing tumor; NEL, nonenhancing lesion; nCBV, normalized cerebral blood volume; nCBF, normalized cerebral blood flow, CMRO_2_, cerebral metabolic rate of oxygen; COV, coefficient of variation; CTH, capillary transit time heterogeneity; MTT, mean transit time; OEF, oxygen extraction fraction; TTP, time to peak; ADC, apparent diffusion coefficient.

*indicates statistical significance.

**Supplementary Table 3. Selected Features of the Concordant Model and the Best Model**

| **Feature** | **AUC** | **Cutoff** | **Sensitivity** | **Specificity** | **Accuracy** |
| --- | --- | --- | --- | --- | --- |
| **IDH mutation** |  |  |  |  |  |
| **Concordant model (n = 49)** |  |  |  |  |  |
| NEL_Shape _Elongation_ | 0.633 (0.563–0.704) | 0.166 | 90.8% | 28.8% | 43.9% |
| NEL_Shape _Flatness_ | 0.68 (0.614–0.746) | 0.208 | 82.9% | 49.6% | 57.7% |
| CET_Shape _MajorAxisLength_ | 0.545 (0.475–0.615) | 0.221 | 85.5% | 28.4% | 42.3% |
| NEL_Shape _Maximum3DDiameter_ | 0.625 (0.552–0.698) | 0.224 | 69.7% | 53.4% | 57.4% |
| CET_ADC_Firstorder _10Percentile_ | 0.707 (0.637–0.776) | 0.232 | 69.7% | 65.3% | 66.3% |
| NEL_CBV_Firstorder _90Percentile_ | 0.522 (0.449–0.595) | 0.232 | 80.3% | 26.7% | 39.7% |
| CET_OEF_Firstorder _Kurtosis_ | 0.523 (0.448–0.597) | 0.244 | 40.8% | 66.9% | 39.4% |
| NEL_CBF_Firstorder _Maximum_ | 0.525 (0.45–0.599) | 0.245 | 60.5% | 54.2% | 44.2% |
| CET_CBV_Firstorder _MeanAbsoluteDeviation_ | 0.716 (0.656–0.776) | 0.225 | 80.3% | 55.9% | 61.9% |
| NEL_ADC_Firstorder _Median_ | 0.629 (0.563–0.694) | 0.235 | 72.4% | 56.8% | 60.6% |
| NEL_MTT_Firstorder _Minimum_ | 0.463 (0.386–0.541) | 0.246 | 53.9% | 50.4% | 48.7% |
| NEL_CBF_Firstorder _Range_ | 0.525 (0.451–0.599) | 0.245 | 60.5% | 54.2% | 44.2% |
| NEL_rCMRO2_Firstorder _Skewness_ | 0.573 (0.505–0.641) | 0.204 | 90.8% | 27.5% | 42.9% |
| CET_COV_Firstorder _Skewness_ | 0.569 (0.489–0.65) | 0.251 | 46.1% | 72.9% | 66.3% |
| CET_ADC_Firstorder _Skewness_ | 0.592 (0.516–0.668) | 0.300 | 34.2% | 84.3% | 72.1% |
| NEL_rCMRO2_GLCM _Contrast_ | 0.527 (0.452–0.603) | 0.256 | 30.3% | 77.1% | 65.7% |
| NEL_CBF_GLCM _Correlation_ | 0.505 (0.451–0.559) | 0.256 | 17.1% | 92.4% | 74.0% |
| NEL_CTH_GLCM _Correlation_ | 0.516 (0.439–0.592) | 0.256 | 23.7% | 86.0% | 70.8% |
| NEL_MTT_GLCM _Idmn_ | 0.534 (0.462–0.606) | 0.238 | 78.9% | 33.1% | 44.2% |
| CET_TTP_GLCM _Idmn_ | 0.534 (0.454–0.615) | 0.245 | 36.8% | 75.8% | 66.3% |
| NEL_ADC_GLCM _InverseVariance_ | 0.614 (0.546–0.682) | 0.236 | 75.0% | 47.9% | 54.5% |
| CET_MTT_GLCM _InverseVariance_ | 0.509 (0.428–0.59) | 0.235 | 31.6% | 79.7% | 32.1% |
| CET_rCMRO2_GLCM _MCC_ | 0.536 (0.458–0.614) | 0.228 | 63.2% | 53.0% | 55.4% |
| NEL_ADC_GLCM _MaximumProbability_ | 0.528 (0.459–0.596) | 0.241 | 71.1% | 44.1% | 49.4% |
| CET_rCMRO2_GLCM _SumSquares_ | 0.62 (0.551–0.689) | 0.215 | 84.2% | 41.9% | 52.2% |
| CET_OEF_GLDM _DependenceEntropy_ | 0.635 (0.555–0.714) | 0.277 | 52.6% | 80.5% | 73.7% |
| CET_CBV_GLDM _DependenceNonUniformity_ | 0.603 (0.516–0.691) | 0.282 | 39.5% | 93.6% | 80.4% |
| NEL_COV_GLDM _DependenceNonUniformity_ | 0.556 (0.484–0.627) | 0.229 | 78.9% | 36.0% | 46.5% |
| NEL_CTH_GLDM _DependenceNonUniformity_ | 0.549 (0.478–0.621) | 0.232 | 77.6% | 36.4% | 46.5% |
| CET_OEF_GLDM _DependenceVariance_ | 0.541 (0.469–0.612) | 0.235 | 81.6% | 33.1% | 44.9% |
| CET_TTP_GLRLM _RunLengthNonUniformity_ | 0.597 (0.522–0.672) | 0.248 | 67.1% | 55.5% | 58.3% |
| NEL_MTT_GLRLM _RunLengthNonUniformityNormalized_ | 0.459 (0.383–0.535) | 0.203 | 7.9% | 98.7% | 23.4% |
| NEL_CBV_GLRLM _RunLengthNonUniformityNormalized_ | 0.615 (0.521–0.708) | 0.433 | 50.0% | 93.2% | 82.7% |
| NEL_OEF_GLRLM _RunVariance_ | 0.468 (0.388–0.548) | 0.272 | 11.8% | 97.9% | 76.9% |
| NEL_TTP_GLSZM _GrayLevelNonUniformity_ | 0.564 (0.487–0.642) | 0.246 | 38.2% | 75.0% | 66.0% |
| NEL_CBF_GLSZM _GrayLevelNonUniformity_ | 0.623 (0.553–0.692) | 0.252 | 80.3% | 48.7% | 56.4% |
| NEL_TTP_GLSZM _GrayLevelNonUniformityNormalized_ | 0.571 (0.491–0.651) | 0.257 | 56.6% | 61.0% | 59.9% |
| CET_TTP_GLSZM _GrayLevelNonUniformityNormalized_ | 0.609 (0.528–0.689) | 0.298 | 40.8% | 84.7% | 74.0% |
| NEL_ADC_GLSZM _HighGrayLevelZoneEmphasis_ | 0.609 (0.538–0.68) | 0.192 | 90.8% | 28.4% | 43.6% |
| NEL_TTP_GLSZM _LargeAreaLowGrayLevelEmphasis_ | 0.536 (0.459–0.613) | 0.234 | 48.7% | 63.6% | 59.9% |
| CET_TTP_GLSZM _SizeZoneNonUniformityNormalized_ | 0.578 (0.503–0.653) | 0.192 | 80.3% | 35.6% | 46.5% |
| CET_CBF_GLSZM _SmallAreaHighGrayLevelEmphasis_ | 0.604 (0.528–0.679) | 0.271 | 28.9% | 90.3% | 75.3% |
| NEL_ADC_GLSZM _ZoneEntropy_ | 0.479 (0.395–0.564) | 0.242 | 38.2% | 74.2% | 34.6% |
| CET_MTT_NGTDM _Coarseness_ | 0.543 (0.468–0.618) | 0.254 | 44.7% | 69.5% | 36.5% |
| CET_TTP_NGTDM _Coarseness_ | 0.571 (0.492–0.649) | 0.232 | 42.1% | 73.7% | 66.0% |
| CET_rCMRO2_NGTDM _Strength_ | 0.517 (0.442–0.592) | 0.252 | 27.6% | 81.4% | 68.3% |
| NEL_CBF_NGTDM _Strength_ | 0.613 (0.541–0.684) | 0.235 | 68.4% | 53.0% | 56.7% |
| CBF Intersection | 0.669 (0.596–0.742) | 0.293 | 57.9% | 73.3% | 69.6% |
| CMRO2 Intersection | 0.674 (0.599–0.748) | 0.257 | 69.7% | 66.5% | 67.3% |
| **Best model (n = 30)** |  |  |  |  |  |
| NEL_Shape _Flatness_ | 0.68 (0.614–0.746) | 0.208 | 82.9% | 49.6% | 57.7% |
| NEL_ADC_Firstorder _10Percentile_ | 0.624 (0.556–0.692) | 0.269 | 53.9% | 68.6% | 65.1% |
| NEL_CMRO2_Firstorder _10Percentile_ | 0.559 (0.484–0.634) | 0.243 | 59.2% | 58.5% | 58.7% |
| NEL_ADC_Firstorder _Maximum_ | 0.62 (0.55–0.69) | 0.248 | 63.2% | 57.6% | 59.0% |
| CET_nCBV_Firstorder _RobustMeanAbsoluteDeviation_ | 0.733 (0.674–0.793) | 0.226 | 80.3% | 58.1% | 63.5% |
| CET_ADC_Firstorder _RootMeanSquared_ | 0.67 (0.6–0.74) | 0.298 | 44.7% | 81.8% | 72.8% |
| NEL_CMRO2_Firstorder _Skewness_ | 0.573 (0.505–0.641) | 0.204 | 90.8% | 27.5% | 42.9% |
| NEL_CTH_Firstorder _TotalEnergy_ | 0.586 (0.508–0.664) | 0.237 | 42.1% | 78.4% | 30.4% |
| NEL_OEF_GLCM _ClusterShade_ | 0.583 (0.519–0.647) | 0.232 | 86.8% | 25.8% | 40.7% |
| NEL_CTH_GLCM _Correlation_ | 0.516 (0.439–0.592) | 0.256 | 23.7% | 86.0% | 70.8% |
| CET_CMRO2_GLCM _Idmn_ | 0.696 (0.628–0.763) | 0.281 | 67.1% | 70.3% | 69.6% |
| NEL_ADC_GLCM _InverseVariance_ | 0.614 (0.546–0.682) | 0.236 | 75.0% | 47.9% | 54.5% |
| CET_ADC_GLCM _MCC_ | 0.546 (0.469–0.623) | 0.283 | 25.0% | 86.9% | 71.8% |
| CET_TTP_GLRLM _LongRunLowGrayLevelEmphasis_ | 0.613 (0.529–0.697) | 0.250 | 40.8% | 87.3% | 76.0% |
| NEL_ADC_GLSZM _GrayLevelVariance_ | 0.667 (0.6–0.734) | 0.225 | 81.6% | 49.2% | 57.1% |
| NEL_ADC_GLSZM _HighGrayLevelZoneEmphasis_ | 0.609 (0.538–0.68) | 0.192 | 90.8% | 28.4% | 43.6% |
| NEL_TTP_GLSZM _LargeAreaLowGrayLevelEmphasis_ | 0.536 (0.459–0.613) | 0.234 | 48.7% | 63.6% | 59.9% |
| CET_MTT_GLSZM _SmallAreaLowGrayLevelEmphasis_ | 0.606 (0.53–0.681) | 0.268 | 50.0% | 69.5% | 64.7% |
| CET_MTT_GLSZM _ZoneVariance_ | 0.6 (0.524–0.677) | 0.241 | 30.3% | 93.2% | 22.1% |
| NEL_nCBV_GLSZM _ZoneVariance_ | 0.537 (0.458–0.616) | 0.229 | 34.2% | 79.2% | 31.7% |
| CET_nCBF_GLDM _DependenceEntropy_ | 0.785 (0.73–0.839) | 0.191 | 85.5% | 66.5% | 71.2% |
| NEL_nCBF_GLDM _DependenceEntropy_ | 0.838 (0.785–0.891) | 0.388 | 64.5% | 91.5% | 84.9% |
| NEL_nCBF_GLDM _DependenceVariance_ | 0.805 (0.742–0.869) | 0.200 | 73.7% | 80.5% | 78.8% |
| NEL_nCBV_GLDM _DependenceVariance_ | 0.802 (0.738–0.867) | 0.196 | 73.7% | 79.2% | 77.9% |
| CET_CTH_GLDM _SmallDependenceHighGrayLevelEmphasis_ | 0.586 (0.503–0.669) | 0.233 | 27.6% | 97.0% | 19.9% |
| CET_MTT_NGTDM _Coarseness_ | 0.543 (0.468–0.618) | 0.254 | 44.7% | 69.5% | 36.5% |
| NEL_nCBF_NGTDM _Contrast_ | 0.633 (0.568–0.698) | 0.215 | 88.2% | 38.1% | 50.3% |
| CBF Intersection | 0.669 (0.596–0.742) | 0.293 | 57.9% | 73.3% | 69.6% |
| CBV Intersection | 0.695 (0.621–0.769) | 0.296 | 60.5% | 76.3% | 72.4% |
| CTH Intersection | 0.622 (0.543–0.7) | 0.305 | 40.8% | 86.0% | 75.0% |
| **EGFR amplification** |  |  |  |  |  |
| **Concordant model (n = 7)** |  |  |  |  |  |
| NEL_Shape _Sphericity_ | 0.588 (0.509–0.667) | 0.270 | 74.3% | 45.2% | 53.8% |
| CET_nCBV_Firstorder _90Percentile_ | 0.642 (0.566–0.718) | 0.287 | 60.0% | 63.9% | 62.7% |
| CET_ADC_Firstorder _Maximum_ | 0.592 (0.517–0.668) | 0.251 | 90.0% | 33.1% | 50.0% |
| NEL_nCBF_Firstorder _Skewness_ | 0.623 (0.548–0.699) | 0.287 | 71.4% | 50.0% | 56.4% |
| NEL_CMRO2_GLCM _Autocorrelation_ | 0.638 (0.56–0.717) | 0.280 | 60.0% | 65.1% | 63.6% |
| CET_OEF_GLDM _SmallDependenceLowGrayLevelEmphasis_ | 0.552 (0.466–0.638) | 0.298 | 48.6% | 70.5% | 64.0% |
| NEL_ADC_GLSZM _GrayLevelVariance_ | 0.551 (0.473–0.628) | 0.280 | 81.4% | 31.9% | 46.6% |
| **Best model (n = 19)** |  |  |  |  |  |
| NEL_nCBF_Firstorder _Kurtosis_ | 0.641 (0.566–0.716) | 0.307 | 74.3% | 50.0% | 57.2% |
| CET_nCBV_Firstorder _Median_ | 0.672 (0.596–0.748) | 0.268 | 68.6% | 61.4% | 63.6% |
| CET_CMRO2_Firstorder _Minimum_ | 0.561 (0.478–0.644) | 0.261 | 71.4% | 45.2% | 53.0% |
| CET_nCBF_Firstorder _Minimum_ | 0.536 (0.449–0.622) | 0.291 | 35.7% | 78.9% | 66.1% |
| CET_nCBV_Firstorder _RobustMeanAbsoluteDeviation_ | 0.63 (0.551–0.709) | 0.398 | 30.0% | 91.0% | 72.9% |
| NEL_nCBV_Firstorder _RobustMeanAbsoluteDeviation_ | 0.654 (0.578–0.729) | 0.249 | 75.7% | 49.4% | 57.2% |
| NEL_MTT_Firstorder _Skewness_ | 0.552 (0.467–0.638) | 0.357 | 30.0% | 88.6% | 71.2% |
| NEL_CMRO2_Firstorder _TotalEnergy_ | 0.606 (0.524–0.688) | 0.288 | 55.7% | 68.1% | 64.4% |
| CET_CMRO2_Firstorder _Variance_ | 0.633 (0.553–0.714) | 0.307 | 57.1% | 69.9% | 66.1% |
| CET_MTT_GLCM _ClusterShade_ | 0.542 (0.462–0.621) | 0.305 | 34.3% | 78.9% | 34.3% |
| CET_CMRO2_GLCM _ClusterTendency_ | 0.632 (0.553–0.711) | 0.294 | 60.0% | 68.7% | 66.1% |
| CET_CMRO2_GLCM _Idn_ | 0.66 (0.585–0.735) | 0.322 | 55.7% | 74.7% | 69.1% |
| CET_COV_GLCM _MCC_ | 0.523 (0.451–0.594) | 0.376 | 15.7% | 95.2% | 71.6% |
| NEL_nCBV_GLSZM _SmallAreaEmphasis_ | 0.581 (0.502–0.659) | 0.261 | 77.1% | 42.2% | 52.5% |
| CET_CMRO2_GLDM _GrayLevelVariance_ | 0.634 (0.553–0.714) | 0.307 | 57.1% | 70.5% | 66.5% |
| CET_OEF_GLDM _SmallDependenceLowGrayLevelEmphasis_ | 0.552 (0.466–0.638) | 0.298 | 48.6% | 70.5% | 64.0% |
| CET_nCBF_NGTDM _Coarseness_ | 0.508 (0.421–0.594) | 0.266 | 51.4% | 61.4% | 41.5% |
| NEL_ADC_NGTDM _Strength_ | 0.55 (0.473–0.626) | 0.289 | 80.0% | 36.7% | 49.6% |
| CET_nCBV_NGTDM _Strength_ | 0.604 (0.531–0.678) | 0.296 | 92.9% | 28.9% | 47.9% |

IDH, isocitrate dehydrogenase; EGFR, epidermal growth factor receptor; CET, contrast-enhancing tumor; NEL, nonenhancing lesion; nCBV, normalized cerebral blood volume; nCBF, normalized cerebral blood flow, CMRO_2_ , cerebral metabolic rate of oxygen; COV, coefficient of variation; CTH, capillary transit time heterogeneity; MTT, mean transit time; OEF, oxygen extraction fraction; TTP, time to peak; ADC, apparent diffusion coefficient; AUC, area under the receiver operating characteristic curve.
