# Supplementary material for: Imaging‐Based Molecular Characterization of Adult‐Type Diffuse Glioma Using Diffusion and Perfusion MRI in Pre‐ and Post‐Treatment Stage Considering Spatial and Temporal Heterogeneity
Source: J Magn Reson Imaging. 2025 Apr 8;62(2):468–79. doi: 10.1002/jmri.29781 (PMC12276639; doi:10.1002/jmri.29781)
Supplement: Supplementary file 2 — Data S2. [file JMRI-62-468-s002.pdf]

Supplementary Material 3. Intraclass Coefficient (ICC) of Temporally Stable MRI Features.

Note: Overlapping features are highlighted.

IDH mutant	ICC (95% condifence intervals)
shape_Elongation_NEL	0.81 (0.04–0.96)
shape_Flatness_NEL	0.6 (-1.02–0.92)
shape_MajorAxisLength_CET	0.6 (-1–0.92)
shape_MinorAxisLength_NEL	0.62 (-0.91–0.92)
shape_SurfaceVolumeRatio_NEL	0.73 (-0.33–0.95)
firstorder_10Percentile_NEL_MTT	0.74 (-0.31–0.95)
firstorder_10Percentile_CET_CMRO2	0.55 (-1.24–0.91)
firstorder_10Percentile_CET_ADC	0.94 (0.72–0.99)
firstorder_90Percentile_CET_CMRO2	0.57 (-1.14–0.91)
firstorder_90Percentile_CET_ADC	0.56 (-1.21–0.91)
firstorder_90Percentile_NEL_CBF	0.56 (-1.2–0.91)
firstorder_90Percentile_NEL_CBV	0.54 (-1.28–0.91)
firstorder_Energy_NEL_OEF	0.58 (-1.11–0.92)
firstorder_Entropy_NEL_ADC	0.6 (-0.99–0.92)
firstorder_Entropy_CET_OEF	0.53 (-1.35–0.91)
firstorder_InterquartileRange_CET_CMRO2	0.54 (-1.31–0.91)
firstorder_InterquartileRange_CET_ADC	0.85 (0.26–0.97)
firstorder_InterquartileRange_NEL_CBF	0.54 (-1.31–0.91)
firstorder_InterquartileRange_NEL_CBV	0.64 (-0.81–0.93)
firstorder_Kurtosis_NEL_OEF	0.77 (-0.12–0.95)
firstorder_Kurtosis_NEL_CMRO2	0.71 (-0.45–0.94)
firstorder_Kurtosis_CET_COV	0.73 (-0.36–0.95)
firstorder_Kurtosis_CET_OEF	0.64 (-0.8–0.93)
firstorder_Kurtosis_NEL_CBF	0.61 (-0.95–0.92)
firstorder_Kurtosis_NEL_CBV	0.76 (-0.22–0.95)
firstorder_Maximum_NEL_CBF	0.51 (-1.45–0.9)
firstorder_Maximum_CET_CBV	0.62 (-0.91–0.92)
firstorder_MeanAbsoluteDeviation_CET_CMRO2	0.57 (-1.17–0.91)
firstorder_MeanAbsoluteDeviation_CET_ADC	0.7 (-0.5–0.94)
firstorder_MeanAbsoluteDeviation_NEL_CBF	0.51 (-1.43–0.9)
firstorder_MeanAbsoluteDeviation_NEL_CBV	0.56 (-1.21–0.91)
firstorder_MeanAbsoluteDeviation_CET_CBV	0.5 (-1.48–0.9)
firstorder_Mean_CET_CMRO2	0.59 (-1.06–0.92)
firstorder_Mean_CET_ADC	0.82 (0.11–0.96)
firstorder_Median_NEL_MTT	0.51 (-1.44–0.9)
firstorder_Median_NEL_ADC	0.63 (-0.85–0.93)
firstorder_Median_CET_CMRO2	0.63 (-0.84–0.93)
firstorder_Median_CET_ADC	0.76 (-0.22–0.95)
firstorder_Minimum_CET_ADC	0.63 (-0.85–0.93)
firstorder_Range_NEL_CBF	0.51 (-1.45–0.9)
firstorder_Range_CET_CBV	0.68 (-0.6–0.94)
firstorder_RobustMeanAbsoluteDeviation_CET_CMRO2	0.55 (-1.27–0.91)
firstorder_RobustMeanAbsoluteDeviation_CET_ADC	0.8 (0.02–0.96)
firstorder_RobustMeanAbsoluteDeviation_NEL_CBF	0.55 (-1.27–0.91)
firstorder_RobustMeanAbsoluteDeviation_NEL_CBV	0.64 (-0.82–0.93)
firstorder_RootMeanSquared_CET_CMRO2	0.58 (-1.1–0.92)
firstorder_RootMeanSquared_CET_ADC	0.79 (-0.05–0.96)
firstorder_Skewness_NEL_CMRO2	0.72 (-0.41–0.94)
firstorder_Skewness_CET_COV	0.75 (-0.23–0.95)
firstorder_Skewness_CET_ADC	0.72 (-0.39–0.94)
firstorder_Skewness_NEL_CBF	0.56 (-1.17–0.91)
firstorder_Skewness_NEL_CBV	0.76 (-0.2–0.95)
firstorder_Skewness_NEL_COV	0.66 (-0.72–0.93)
firstorder_TotalEnergy_NEL_OEF	0.58 (-1.12–0.92)
firstorder_Variance_NEL_CMRO2	0.63 (-0.86–0.93)
firstorder_Variance_NEL_ADC	0.51 (-1.46–0.9)
firstorder_Variance_CET_CMRO2	0.66 (-0.68–0.93)
firstorder_Variance_CET_ADC	0.66 (-0.68–0.93)
firstorder_Variance_CET_CBV	0.53 (-1.33–0.91)
glcm_Autocorrelation_CET_CMRO2	0.5 (-1.48–0.9)
glcm_ClusterShade_CET_ADC	0.58 (-1.1–0.92)
glcm_ClusterTendency_NEL_CMRO2	0.55 (-1.25–0.91)
glcm_ClusterTendency_NEL_ADC	0.72 (-0.42–0.94)
glcm_ClusterTendency_CET_CMRO2	0.68 (-0.62–0.94)
glcm_Contrast_NEL_CMRO2	0.56 (-1.18–0.91)
glcm_Correlation_CET_TTP	0.55 (-1.25–0.91)
glcm_Correlation_NEL_CBF	0.82 (0.1–0.96)
glcm_Correlation_NEL_CTH	0.78 (-0.12–0.96)
glcm_DifferenceEntropy_CET_OEF	0.59 (-1.04–0.92)
glcm_DifferenceVariance_NEL_CMRO2	0.59 (-1.02–0.92)
glcm_DifferenceVariance_NEL_ADC	0.51 (-1.47–0.9)
glcm_Idmn_NEL_MTT	0.5 (-1.49–0.9)
glcm_Idmn_NEL_TTP	0.83 (0.15–0.97)
glcm_Idmn_CET_TTP	0.67 (-0.67–0.93)
glcm_Idn_NEL_MTT	0.6 (-1.02–0.92)
glcm_Idn_NEL_TTP	0.75 (-0.25–0.95)
glcm_Imc1_NEL_TTP	0.67 (-0.63–0.93)
glcm_Imc2_NEL_ADC	0.62 (-0.88–0.92)
glcm_InverseVariance_NEL_MTT	0.55 (-1.25–0.91)
glcm_InverseVariance_NEL_OEF	0.53 (-1.34–0.91)
glcm_InverseVariance_NEL_TTP	0.66 (-0.7–0.93)
glcm_InverseVariance_CET_MTT	0.74 (-0.29–0.95)
glcm_InverseVariance_CET_TTP	0.56 (-1.22–0.91)
glcm_JointAverage_NEL_ADC	0.55 (-1.26–0.91)
glcm_JointEnergy_NEL_ADC	0.59 (-1.07–0.92)
glcm_JointEntropy_NEL_ADC	0.52 (-1.39–0.9)
glcm_JointEntropy_CET_OEF	0.67 (-0.63–0.93)

IDH wild	ICC (95% condifence intervals)
shape_Maximum2DDiameterRow_NEL	0.51 (-0.03–0.77)
shape_Maximum2DDiameterSlice_NEL	0.55 (0.05–0.78)
shape_Maximum3DDiameter_NEL	0.54 (0.04–0.78)
firstorder_10Percentile_CET_ADC	0.57 (0.1–0.8)
firstorder_Energy_NEL_CBF	0.6 (0.15–0.81)
firstorder_Kurtosis_NEL_CMRO2	0.51 (-0.03–0.77)
firstorder_Kurtosis_CET_ADC	0.69 (0.35–0.85)
firstorder_Maximum_CET_CBF	0.73 (0.43–0.87)
firstorder_Maximum_NEL_CBF	0.62 (0.19–0.82)
firstorder_Median_CET_ADC	0.56 (0.08–0.79)
firstorder_Minimum_NEL_MTT	0.52 (-0.01–0.77)
firstorder_Minimum_NEL_CMRO2	0.7 (0.38–0.86)
firstorder_Range_CET_CBF	0.72 (0.42–0.87)
firstorder_Range_NEL_CBF	0.62 (0.2–0.82)
firstorder_Skewness_NEL_CMRO2	0.6 (0.16–0.81)
firstorder_TotalEnergy_NEL_CBF	0.51 (-0.02–0.77)
glcm_Correlation_CET_CBF	0.52 (-0.01–0.77)
glcm_Id_NEL_ADC	0.56 (0.07–0.79)
glcm_Idm_NEL_ADC	0.6 (0.16–0.81)
glcm_Idn_NEL_TTP	0.56 (0.08–0.79)
glcm_Imc1_NEL_TTP	0.64 (0.25–0.83)
glcm_Imc2_NEL_TTP	0.59 (0.14–0.8)
glcm_InverseVariance_NEL_TTP	0.52 (-0.02–0.77)
glcm_InverseVariance_NEL_ADC	0.54 (0.03–0.78)
glcm_JointEnergy_NEL_TTP	0.51 (-0.02–0.77)
gldm_DependenceNonUniformity_CET_TTP	0.77 (0.52–0.89)
gldm_DependenceNonUniformity_CET_CBV	0.58 (0.11–0.8)
gldm_DependenceNonUniformity_NEL_COV	0.73 (0.44–0.87)
gldm_DependenceNonUniformity_NEL_CTH	0.75 (0.47–0.88)
gldm_DependenceNonUniformityNormalized_NEL_ADC	0.71 (0.39–0.86)
gldm_DependenceVariance_NEL_ADC	0.58 (0.11–0.8)
gldm_GrayLevelNonUniformity_NEL_ADC	0.6 (0.17–0.81)
gldm_LargeDependenceEmphasis_NEL_ADC	0.67 (0.3–0.84)
gldm_LowGrayLevelEmphasis_NEL_TTP	0.56 (0.08–0.79)
gldm_SmallDependenceEmphasis_NEL_ADC	0.76 (0.5–0.89)
gldm_SmallDependenceLowGrayLevelEmphasis_NEL_OEF	0.53 (0.01–0.77)
glrlm_GrayLevelNonUniformity_NEL_ADC	0.6 (0.16–0.81)
glrlm_GrayLevelNonUniformityNormalized_NEL_TTP	0.56 (0.07–0.79)
glrlm_LongRunEmphasis_NEL_ADC	0.73 (0.43–0.87)
glrlm_LowGrayLevelRunEmphasis_NEL_TTP	0.57 (0.1–0.8)
glrlm_RunLengthNonUniformity_NEL_TTP	0.58 (0.12–0.8)
glrlm_RunLengthNonUniformityNormalized_NEL_ADC	0.69 (0.34–0.85)
glrlm_RunPercentage_NEL_ADC	0.71 (0.4–0.86)
glrlm_RunVariance_NEL_ADC	0.73 (0.43–0.87)
glrlm_ShortRunEmphasis_NEL_ADC	0.69 (0.35–0.85)
glrlm_ShortRunLowGrayLevelEmphasis_NEL_TTP	0.51 (-0.02–0.77)
glrlm_ShortRunLowGrayLevelEmphasis_NEL_COV	0.51 (-0.03–0.77)
glszm_GrayLevelNonUniformity_NEL_TTP	0.73 (0.43–0.87)
glszm_GrayLevelNonUniformity_CET_OEF	0.59 (0.14–0.81)
glszm_GrayLevelNonUniformity_NEL_CBF	0.53 (0.01–0.78)
glszm_GrayLevelNonUniformityNormalized_CET_CBF	0.52 (-0.01–0.77)
glszm_GrayLevelNonUniformityNormalized_NEL_TTP	0.6 (0.16–0.81)
glszm_GrayLevelNonUniformityNormalized_CET_TTP	0.61 (0.18–0.81)
glszm_GrayLevelVariance_CET_CBF	0.52 (-0.01–0.77)
glszm_LargeAreaEmphasis_NEL_TTP	0.59 (0.13–0.8)
glszm_LargeAreaLowGrayLevelEmphasis_NEL_TTP	0.61 (0.18–0.81)
glszm_LowGrayLevelZoneEmphasis_CET_TTP	0.56 (0.08–0.79)
glszm_SizeZoneNonUniformityNormalized_NEL_ADC	0.77 (0.51–0.89)
glszm_SizeZoneNonUniformityNormalized_NEL_CBF	0.6 (0.17–0.81)
glszm_SizeZoneNonUniformityNormalized_NEL_CBV	0.58 (0.12–0.8)
glszm_SmallAreaEmphasis_NEL_ADC	0.77 (0.52–0.89)
glszm_SmallAreaHighGrayLevelEmphasis_CET_CBF	0.58 (0.13–0.8)
glszm_ZoneEntropy_NEL_TTP	0.55 (0.05–0.79)
glszm_ZoneEntropy_NEL_CBF	0.74 (0.46–0.88)
glszm_ZoneEntropy_NEL_CBV	0.66 (0.28–0.84)
glszm_ZonePercentage_NEL_ADC	0.76 (0.5–0.89)
glszm_ZoneVariance_NEL_CBV	0.52 (0–0.77)
ngtdm_Coarseness_NEL_CBV	0.67 (0.3–0.84)
ngtdm_Coarseness_CET_CBV	0.67 (0.3–0.84)

glcm_MCC_NEL_ADC	0.51 (-1.47–0.9)
glcm_MCC_CET_CTH	0.83 (0.14–0.97)
glcm_MCC_CET_CMRO2	0.52 (-1.39–0.9)
glcm_MCC_CET_TTP	0.72 (-0.38–0.94)
glcm_MCC_NEL_CBF	0.86 (0.28–0.97)
glcm_MCC_NEL_CTH	0.8 (-0.02–0.96)
glcm_MaximumProbability_NEL_ADC	0.56 (-1.18–0.91)
glcm_SumAverage_NEL_ADC	0.55 (-1.26–0.91)
glcm_SumEntropy_NEL_ADC	0.64 (-0.81–0.93)
glcm_SumEntropy_CET_OEF	0.64 (-0.8–0.93)
glcm_SumSquares_NEL_CMRO2	0.57 (-1.14–0.91)
glcm_SumSquares_CET_CMRO2	0.61 (-0.97–0.92)
glcm_SumSquares_CET_ADC	0.58 (-1.08–0.92)
gldm_DependenceEntropy_CET_OEF	0.57 (-1.16–0.91)
gldm_DependenceNonUniformityNormalized_NEL_ADC	0.69 (-0.57–0.94)
gldm_DependenceVariance_NEL_ADC	0.67 (-0.66–0.93)
gldm_DependenceVariance_CET_OEF	0.6 (-1–0.92)
gldm_GrayLevelNonUniformity_NEL_MTT	0.59 (-1.06–0.92)
gldm_GrayLevelVariance_NEL_CMRO2	0.63 (-0.86–0.93)
gldm_GrayLevelVariance_NEL_ADC	0.51 (-1.45–0.9)
gldm_GrayLevelVariance_CET_CMRO2	0.66 (-0.68–0.93)
gldm_GrayLevelVariance_CET_ADC	0.66 (-0.68–0.93)
gldm_LargeDependenceEmphasis_NEL_MTT	0.69 (-0.53–0.94)
gldm_LargeDependenceEmphasis_NEL_OEF	0.53 (-1.34–0.91)
gldm_LargeDependenceEmphasis_NEL_CMRO2	0.53 (-1.34–0.91)
gldm_LargeDependenceEmphasis_NEL_ADC	0.71 (-0.46–0.94)
gldm_LargeDependenceEmphasis_NEL_CBF	0.7 (-0.51–0.94)
gldm_LargeDependenceEmphasis_NEL_CBV	0.72 (-0.38–0.94)
gldm_LargeDependenceHighGrayLevelEmphasis_NEL_CBF	0.71 (-0.46–0.94)
gldm_LargeDependenceHighGrayLevelEmphasis_NEL_CBV	0.72 (-0.38–0.94)
gldm_LargeDependenceLowGrayLevelEmphasis_NEL_MTT	0.69 (-0.54–0.94)
gldm_LargeDependenceLowGrayLevelEmphasis_NEL_OEF	0.53 (-1.34–0.91)
gldm_LargeDependenceLowGrayLevelEmphasis_NEL_CMRO2	0.53 (-1.33–0.91)
gldm_LargeDependenceLowGrayLevelEmphasis_NEL_CBF	0.7 (-0.52–0.94)
gldm_LargeDependenceLowGrayLevelEmphasis_NEL_CBV	0.72 (-0.38–0.94)
gldm_SmallDependenceEmphasis_NEL_ADC	0.76 (-0.2–0.95)
gldm_SmallDependenceEmphasis_NEL_CBF	0.59 (-1.05–0.92)
gldm_SmallDependenceEmphasis_NEL_CBV	0.89 (0.47–0.98)
gldm_SmallDependenceHighGrayLevelEmphasis_NEL_ADC	0.63 (-0.83–0.93)
gldm_SmallDependenceHighGrayLevelEmphasis_NEL_CBV	0.89 (0.47–0.98)
gldm_SmallDependenceLowGrayLevelEmphasis_NEL_TTP	0.84 (0.18–0.97)
gldm_SmallDependenceLowGrayLevelEmphasis_NEL_CBF	0.8 (0.01–0.96)
gldm_SmallDependenceLowGrayLevelEmphasis_NEL_CBV	0.89 (0.47–0.98)
gldm_SmallDependenceLowGrayLevelEmphasis_NEL_CTH	0.69 (-0.55–0.94)
glrlm_GrayLevelVariance_NEL_CMRO2	0.63 (-0.87–0.93)
glrlm_GrayLevelVariance_NEL_ADC	0.51 (-1.46–0.9)
glrlm_GrayLevelVariance_CET_CMRO2	0.66 (-0.68–0.93)
glrlm_GrayLevelVariance_CET_ADC	0.64 (-0.8–0.93)
glrlm_LongRunEmphasis_NEL_ADC	0.75 (-0.23–0.95)
glrlm_RunEntropy_NEL_CBF	0.82 (0.11–0.96)
glrlm_RunEntropy_NEL_CBV	0.78 (-0.12–0.96)
glrlm_RunLengthNonUniformity_CET_TTP	0.84 (0.18–0.97)
glrlm_RunLengthNonUniformity_NEL_CBF	0.5 (-1.49–0.9)
glrlm_RunLengthNonUniformity_NEL_CBV	0.51 (-1.47–0.9)
glrlm_RunLengthNonUniformityNormalized_NEL_MTT	0.55 (-1.26–0.91)
glrlm_RunLengthNonUniformityNormalized_NEL_ADC	0.68 (-0.58–0.94)
glrlm_RunLengthNonUniformityNormalized_NEL_CBF	0.86 (0.29–0.97)
glrlm_RunLengthNonUniformityNormalized_NEL_CBV	0.88 (0.42–0.98)
glrlm_RunPercentage_NEL_MTT	0.61 (-0.94–0.92)
glrlm_RunPercentage_NEL_ADC	0.72 (-0.38–0.94)
glrlm_RunPercentage_NEL_CBF	0.75 (-0.25–0.95)
glrlm_RunPercentage_NEL_CBV	0.79 (-0.07–0.96)
glrlm_RunVariance_NEL_MTT	0.62 (-0.92–0.92)
glrlm_RunVariance_NEL_OEF	0.57 (-1.13–0.91)
glrlm_RunVariance_NEL_ADC	0.78 (-0.09–0.96)
glrlm_ShortRunEmphasis_NEL_MTT	0.62 (-0.9–0.92)
glrlm_ShortRunEmphasis_NEL_OEF	0.51 (-1.46–0.9)
glrlm_ShortRunEmphasis_NEL_ADC	0.7 (-0.52–0.94)
glrlm_ShortRunEmphasis_NEL_CBF	0.74 (-0.29–0.95)
glrlm_ShortRunEmphasis_NEL_CBV	0.79 (-0.07–0.96)
glrlm_ShortRunHighGrayLevelEmphasis_NEL_CBV	0.79 (-0.07–0.96)
glrlm_ShortRunLowGrayLevelEmphasis_NEL_CBF	0.8 (-0.01–0.96)
glrlm_ShortRunLowGrayLevelEmphasis_NEL_CBV	0.79 (-0.07–0.96)
glszm_GrayLevelNonUniformity_CET_TTP	0.85 (0.24–0.97)
glszm_GrayLevelNonUniformityNormalized_NEL_ADC	0.56 (-1.19–0.91)
glszm_GrayLevelNonUniformityNormalized_CET_MTT	0.72 (-0.42–0.94)
glszm_GrayLevelNonUniformityNormalized_NEL_CBF	0.84 (0.18–0.97)
glszm_GrayLevelVariance_NEL_CMRO2	0.63 (-0.86–0.93)
glszm_GrayLevelVariance_CET_CMRO2	0.65 (-0.77–0.93)
glszm_GrayLevelVariance_NEL_CBF	0.84 (0.18–0.97)
glszm_HighGrayLevelZoneEmphasis_NEL_ADC	0.52 (-1.42–0.9)
glszm_HighGrayLevelZoneEmphasis_NEL_CBF	0.67 (-0.67–0.93)
glszm_LargeAreaEmphasis_NEL_MTT	0.69 (-0.55–0.94)
glszm_LargeAreaHighGrayLevelEmphasis_NEL_MTT	0.69 (-0.55–0.94)
glszm_LargeAreaLowGrayLevelEmphasis_NEL_MTT	0.69 (-0.55–0.94)
glszm_LowGrayLevelZoneEmphasis_NEL_MTT	0.53 (-1.33–0.91)
glszm_LowGrayLevelZoneEmphasis_CET_MTT	0.7 (-0.49–0.94)
glszm_LowGrayLevelZoneEmphasis_NEL_CBF	0.67 (-0.67–0.93)
glszm_SizeZoneNonUniformityNormalized_NEL_TTP	0.64 (-0.8–0.93)
glszm_SizeZoneNonUniformityNormalized_NEL_ADC	0.8 (-0.02–0.96)
glszm_SizeZoneNonUniformityNormalized_CET_TTP	0.55 (-1.25–0.91)
glszm_SizeZoneNonUniformityNormalized_NEL_CTH	0.52 (-1.4–0.9)
glszm_SmallAreaEmphasis_NEL_MTT	0.63 (-0.87–0.93)

glszm_SmallAreaEmphasis_NEL_OEF	0.69 (-0.57–0.94)
glszm_SmallAreaEmphasis_NEL_TTP	0.61 (-0.93–0.92)
glszm_SmallAreaEmphasis_NEL_ADC	0.79 (-0.04–0.96)
glszm_SmallAreaEmphasis_CET_MTT	0.55 (-1.22–0.91)
glszm_SmallAreaEmphasis_NEL_CTH	0.61 (-0.94–0.92)
glszm_SmallAreaHighGrayLevelEmphasis_NEL_ADC	0.66 (-0.69–0.93)
glszm_SmallAreaHighGrayLevelEmphasis_NEL_CBF	0.55 (-1.25–0.91)
glszm_SmallAreaLowGrayLevelEmphasis_CET_TTP	0.61 (-0.96–0.92)
glszm_ZoneEntropy_NEL_ADC	0.65 (-0.75–0.93)
glszm_ZonePercentage_NEL_ADC	0.74 (-0.32–0.95)
ngtdm_Busyness_CET_TTP	0.7 (-0.48–0.94)
ngtdm_Coarseness_CET_MTT	0.77 (-0.13–0.95)
ngtdm_Coarseness_CET_TTP	0.72 (-0.38–0.94)
ngtdm_Coarseness_NEL_CBF	0.77 (-0.13–0.95)
ngtdm_Coarseness_NEL_CBV	0.67 (-0.66–0.93)
ngtdm_Coarseness_CET_CBV	0.67 (-0.66–0.93)
ngtdm_Coarseness_NEL_CTH	0.75 (-0.25–0.95)
ngtdm_Complexity_NEL_ADC	0.8 (0–0.96)
ngtdm_Contrast_NEL_CMRO2	0.54 (-1.3–0.91)
ngtdm_Strength_CET_CMRO2	0.52 (-1.39–0.9)
ngtdm_Strength_NEL_CBF	0.99 (0.96–1)
CBF_Intersection	0.8 (0.02–0.96)
CBF_BD	0.82 (0.08–0.96)
CMRO2_Intersection	0.67 (-0.65–0.93)
CMRO2_BD	0.63 (-0.87–0.93)

EGFR amplification	ICC (95% condifence intervals)
shape_Flatness_CET	0.53 (-2.34–0.93)
shape_MajorAxisLength_NEL	0.64 (-1.56–0.95)
shape_Maximum2DDiameterRow_NEL	0.5 (-2.55–0.93)
shape_Maximum2DDiameterSlice_CET	0.62 (-1.71–0.95)
shape_Maximum2DDiameterSlice_NEL	0.7 (-1.16–0.96)
shape_Maximum3DDiameter_CET	0.53 (-2.34–0.93)
shape_Maximum3DDiameter_NEL	0.61 (-1.78–0.95)
shape_MeshVolume_NEL	0.55 (-2.22–0.94)
shape_MinorAxisLength_NEL	0.51 (-2.5–0.93)
shape_Sphericity_NEL	0.64 (-1.54–0.95)
shape_SurfaceArea_NEL	0.7 (-1.14–0.96)
shape_SurfaceVolumeRatio_CET	0.9 (0.27–0.99)
shape_VoxelVolume_NEL	0.56 (-2.17–0.94)
firstorder_90Percentile_CET_CBF	0.54 (-2.27–0.94)
firstorder_90Percentile_NEL_ADC	0.57 (-2.09–0.94)
firstorder_90Percentile_CET_ADC	0.68 (-1.25–0.96)
firstorder_90Percentile_CET_CBV	0.66 (-1.4–0.95)
firstorder_Energy_CET_CBF	0.53 (-2.37–0.93)
firstorder_Energy_NEL_CBF	0.88 (0.15–0.98)
firstorder_Energy_NEL_CBV	0.61 (-1.76–0.95)
firstorder_Energy_CET_CBV	0.94 (0.54–0.99)
firstorder_Entropy_NEL_CBV	0.67 (-1.36–0.95)
firstorder_Entropy_CET_CBV	0.67 (-1.36–0.95)
firstorder_Kurtosis_NEL_OEF	0.91 (0.39–0.99)
firstorder_Kurtosis_NEL_CMRO2	0.86 (0.03–0.98)
firstorder_Kurtosis_NEL_CBV	0.92 (0.41–0.99)
firstorder_Maximum_CET_CBF	0.96 (0.74–1)
firstorder_Maximum_NEL_ADC	0.51 (-2.54–0.93)
firstorder_Maximum_CET_ADC	0.58 (-2.03–0.94)
firstorder_Maximum_NEL_CBF	0.96 (0.69–0.99)
firstorder_Maximum_NEL_CBV	0.72 (-1.01–0.96)
firstorder_Maximum_CET_CBV	0.72 (-1.01–0.96)
firstorder_MeanAbsoluteDeviation_CET_CBF	0.67 (-1.37–0.95)
firstorder_MeanAbsoluteDeviation_NEL_ADC	0.61 (-1.82–0.94)
firstorder_MeanAbsoluteDeviation_CET_ADC	0.57 (-2.1–0.94)
firstorder_MeanAbsoluteDeviation_NEL_CBF	0.55 (-2.25–0.94)
firstorder_MeanAbsoluteDeviation_CET_CBV	0.79 (-0.51–0.97)
firstorder_Mean_CET_CBF	0.52 (-2.42–0.93)
firstorder_Mean_NEL_ADC	0.64 (-1.54–0.95)
firstorder_Minimum_NEL_CBF	0.52 (-2.42–0.93)
firstorder_Range_CET_CBF	0.97 (0.75–1)
firstorder_Range_NEL_CBF	0.96 (0.7–0.99)
firstorder_Range_NEL_CBV	0.72 (-1.01–0.96)
firstorder_Range_CET_CBV	0.71 (-1.06–0.96)
firstorder_RootMeanSquared_CET_CBF	0.68 (-1.3–0.95)
firstorder_RootMeanSquared_NEL_ADC	0.7 (-1.14–0.96)
firstorder_RootMeanSquared_NEL_CBF	0.62 (-1.69–0.95)
firstorder_Skewness_NEL_CMRO2	0.85 (-0.08–0.98)
firstorder_Skewness_NEL_CBF	0.51 (-2.49–0.93)
firstorder_Skewness_NEL_CBV	0.96 (0.68–0.99)
firstorder_TotalEnergy_CET_CBF	0.52 (-2.42–0.93)
firstorder_TotalEnergy_NEL_CBF	0.89 (0.18–0.98)
firstorder_TotalEnergy_NEL_CBV	0.53 (-2.36–0.93)
firstorder_TotalEnergy_CET_CBV	0.92 (0.42–0.99)
firstorder_Variance_CET_CBF	0.59 (-1.91–0.94)
firstorder_Variance_NEL_ADC	0.83 (-0.24–0.98)
firstorder_Variance_CET_ADC	0.74 (-0.89–0.96)
firstorder_Variance_CET_CBV	0.9 (0.27–0.99)
glcm_ClusterProminence_CET_ADC	0.86 (-0.02–0.98)
glcm_ClusterShade_CET_ADC	0.81 (-0.35–0.97)
glcm_ClusterTendency_NEL_ADC	0.56 (-2.15–0.94)
glcm_ClusterTendency_CET_ADC	0.69 (-1.25–0.96)
glcm_Contrast_NEL_ADC	0.56 (-2.14–0.94)

EGFR non amplification	ICC (95% condifence intervals)
shape_Maximum2DDiameterRow_CET	0.58 (-0.2–0.85)
shape_Maximum2DDiameterRow_NEL	0.51 (-0.4–0.83)
shape_Maximum3DDiameter_CET	0.74 (0.24–0.91)
firstorder_10Percentile_CET_ADC	0.57 (-0.23–0.85)
firstorder_Entropy_NEL_CBV	0.67 (0.05–0.88)
firstorder_Entropy_CET_CBV	0.67 (0.05–0.88)
firstorder_Kurtosis_CET_ADC	0.79 (0.41–0.93)
firstorder_Median_NEL_CMRO2	0.52 (-0.39–0.83)
firstorder_Minimum_NEL_MTT	0.72 (0.21–0.9)
firstorder_Minimum_NEL_CMRO2	0.85 (0.57–0.95)
firstorder_Minimum_CET_ADC	0.63 (-0.07–0.87)
firstorder_Minimum_NEL_CBV	0.54 (-0.32–0.84)
firstorder_Skewness_CET_ADC	0.71 (0.18–0.9)
firstorder_Uniformity_NEL_TTP	0.63 (-0.06–0.87)
firstorder_Variance_NEL_CBF	0.52 (-0.38–0.83)
glcm_Autocorrelation_NEL_CMRO2	0.5 (-0.42–0.83)
glcm_ClusterShade_CET_CMRO2	0.53 (-0.34–0.84)
glcm_DifferenceEntropy_NEL_CBV	0.67 (0.05–0.88)
glcm_DifferenceEntropy_CET_CBV	0.67 (0.05–0.88)
glcm_Id_NEL_ADC	0.56 (-0.26–0.85)
glcm_Idm_NEL_ADC	0.61 (-0.11–0.86)
glcm_Idmn_NEL_CMRO2	0.59 (-0.16–0.86)
glcm_Idmn_NEL_TTP	0.7 (0.14–0.89)
glcm_Idn_NEL_CMRO2	0.59 (-0.17–0.86)
glcm_Idn_NEL_TTP	0.78 (0.36–0.92)
glcm_Imc1_NEL_TTP	0.67 (0.07–0.89)
glcm_Imc2_NEL_TTP	0.7 (0.14–0.89)
glcm_InverseVariance_NEL_TTP	0.75 (0.28–0.91)
glcm_InverseVariance_NEL_ADC	0.54 (-0.3–0.84)
glcm_InverseVariance_CET_TTP	0.59 (-0.19–0.86)
glcm_JointEnergy_NEL_TTP	0.73 (0.22–0.91)
glcm_JointEnergy_CET_TTP	0.56 (-0.26–0.85)
glcm_JointEntropy_NEL_CBV	0.67 (0.05–0.88)
glcm_JointEntropy_CET_CBV	0.67 (0.05–0.88)
glcm_MaximumProbability_NEL_TTP	0.63 (-0.05–0.87)
glcm_SumEntropy_NEL_CBV	0.67 (0.05–0.88)
glcm_SumEntropy_CET_CBV	0.67 (0.05–0.88)
gldm_DependenceNonUniformity_CET_TTP	0.86 (0.6–0.95)
gldm_DependenceNonUniformity_CET_CBV	0.67 (0.05–0.88)
gldm_DependenceNonUniformityNormalized_NEL_ADC	0.78 (0.36–0.92)
gldm_DependenceVariance_NEL_ADC	0.7 (0.13–0.89)
gldm_GrayLevelNonUniformity_CET_ADC	0.51 (-0.39–0.83)
gldm_LargeDependenceEmphasis_NEL_ADC	0.78 (0.36–0.92)
gldm_LowGrayLevelEmphasis_NEL_TTP	0.52 (-0.36–0.83)
gldm_LowGrayLevelEmphasis_CET_TTP	0.51 (-0.4–0.83)
gldm_SmallDependenceEmphasis_NEL_ADC	0.81 (0.45–0.93)
glrlm_GrayLevelNonUniformityNormalized_NEL_TTP	0.65 (-0.01–0.88)
glrlm_LongRunEmphasis_NEL_ADC	0.81 (0.45–0.93)
glrlm_RunLengthNonUniformityNormalized_NEL_ADC	0.79 (0.39–0.93)
glrlm_RunPercentage_NEL_ADC	0.81 (0.45–0.93)
glrlm_RunVariance_NEL_ADC	0.81 (0.46–0.93)
glrlm_RunVariance_CET_CBV	0.54 (-0.33–0.84)
glrlm_ShortRunEmphasis_NEL_ADC	0.79 (0.39–0.93)
glszm_GrayLevelNonUniformity_CET_OEF	0.63 (-0.06–0.87)
glszm_GrayLevelNonUniformityNormalized_NEL_TTP	0.66 (0.03–0.88)
glszm_GrayLevelNonUniformityNormalized_CET_TTP	0.53 (-0.34–0.84)
glszm_LargeAreaEmphasis_NEL_TTP	0.6 (-0.16–0.86)
glszm_LargeAreaHighGrayLevelEmphasis_NEL_TTP	0.51 (-0.39–0.83)
glszm_LargeAreaLowGrayLevelEmphasis_NEL_TTP	0.61 (-0.12–0.86)
glszm_SizeZoneNonUniformityNormalized_NEL_ADC	0.76 (0.31–0.92)
glszm_SizeZoneNonUniformityNormalized_NEL_CTH	0.66 (0.01–0.88)
glszm_SmallAreaEmphasis_NEL_ADC	0.76 (0.32–0.92)
glszm_SmallAreaHighGrayLevelEmphasis_CET_CBF	0.7 (0.14–0.89)

glcm_Contrast_CET_ADC	0.76 (-0.74–0.97)
glcm_Correlation_NEL_TTP	0.78 (-0.56–0.97)
glcm_DifferenceAverage_NEL_ADC	0.51 (-2.47–0.93)
glcm_DifferenceAverage_CET_ADC	0.89 (0.24–0.99)
glcm_DifferenceEntropy_NEL_ADC	0.62 (-1.71–0.95)
glcm_DifferenceEntropy_NEL_CBV	0.67 (-1.36–0.95)
glcm_DifferenceEntropy_CET_CBV	0.67 (-1.36–0.95)
glcm_DifferenceVariance_NEL_ADC	0.63 (-1.66–0.95)
glcm_DifferenceVariance_CET_ADC	0.65 (-1.53–0.95)
glcm_Id_CET_ADC	0.97 (0.81–1)
glcm_Idm_CET_ADC	0.96 (0.74–0.99)
glcm_Idmn_NEL_CMRO2	0.89 (0.19–0.98)
glcm_Idmn_NEL_ADC	0.84 (-0.17–0.98)
glcm_Idmn_CET_ADC	0.77 (-0.67–0.97)
glcm_Idn_NEL_ADC	0.82 (-0.29–0.97)
glcm_Idn_CET_ADC	0.81 (-0.33–0.97)
glcm_Imc1_NEL_TTP	0.78 (-0.57–0.97)
glcm_Imc1_CET_ADC	0.66 (-1.45–0.95)
glcm_Imc2_NEL_TTP	0.55 (-2.23–0.94)
glcm_Imc2_CET_ADC	0.6 (-1.85–0.94)
glcm_InverseVariance_CET_ADC	0.97 (0.79–1)
glcm_JointEntropy_NEL_CBV	0.67 (-1.36–0.95)
glcm_JointEntropy_CET_CBV	0.67 (-1.36–0.95)
glcm_MCC_CET_ADC	0.54 (-2.32–0.93)
glcm_SumEntropy_NEL_CBV	0.67 (-1.36–0.95)
glcm_SumEntropy_CET_CBV	0.67 (-1.36–0.95)
glcm_SumSquares_NEL_ADC	0.75 (-0.81–0.96)
glcm_SumSquares_CET_ADC	0.76 (-0.68–0.97)
gldm_DependenceEntropy_NEL_CBF	0.54 (-2.27–0.94)
gldm_DependenceEntropy_NEL_CBV	0.61 (-1.76–0.95)
gldm_DependenceNonUniformity_NEL_MTT	0.89 (0.22–0.98)
gldm_DependenceNonUniformity_NEL_OEF	0.79 (-0.47–0.97)
gldm_DependenceNonUniformity_NEL_TTP	0.97 (0.79–1)
gldm_DependenceNonUniformity_NEL_ADC	0.53 (-2.34–0.93)
gldm_DependenceNonUniformity_CET_TTP	0.78 (-0.57–0.97)
gldm_DependenceNonUniformity_NEL_CBV	0.51 (-2.52–0.93)
gldm_DependenceNonUniformity_NEL_COV	0.91 (0.32–0.99)
gldm_DependenceNonUniformity_NEL_CTH	0.93 (0.53–0.99)
gldm_DependenceNonUniformityNormalized_CET_ADC	0.98 (0.87–1)
gldm_DependenceNonUniformityNormalized_NEL_CBF	0.59 (-1.94–0.94)
gldm_DependenceNonUniformityNormalized_NEL_CBV	0.61 (-1.82–0.94)
gldm_DependenceVariance_CET_CBF	0.88 (0.14–0.98)
gldm_DependenceVariance_CET_ADC	0.91 (0.35–0.99)
gldm_DependenceVariance_CET_CBV	0.9 (0.28–0.99)
gldm_GrayLevelNonUniformity_NEL_ADC	0.82 (-0.29–0.97)
gldm_GrayLevelNonUniformity_NEL_CBF	0.65 (-1.48–0.95)
gldm_GrayLevelNonUniformity_NEL_CBV	0.66 (-1.44–0.95)
gldm_GrayLevelVariance_NEL_ADC	0.83 (-0.24–0.98)
gldm_GrayLevelVariance_CET_ADC	0.74 (-0.87–0.96)
gldm_LargeDependenceEmphasis_CET_CBF	0.73 (-0.92–0.96)
gldm_LargeDependenceEmphasis_NEL_ADC	0.56 (-2.15–0.94)
gldm_LargeDependenceEmphasis_CET_ADC	0.96 (0.68–0.99)
gldm_LargeDependenceEmphasis_CET_CBV	0.78 (-0.56–0.97)
gldm_LargeDependenceHighGrayLevelEmphasis_CET_CBF	0.75 (-0.79–0.96)
gldm_LargeDependenceHighGrayLevelEmphasis_CET_CBV	0.78 (-0.56–0.97)
gldm_LargeDependenceLowGrayLevelEmphasis_CET_CBF	0.73 (-0.96–0.96)
gldm_LargeDependenceLowGrayLevelEmphasis_NEL_TTP	0.6 (-1.89–0.94)
gldm_LargeDependenceLowGrayLevelEmphasis_CET_TTP	0.54 (-2.28–0.94)
gldm_LargeDependenceLowGrayLevelEmphasis_CET_CBV	0.78 (-0.56–0.97)
gldm_LowGrayLevelEmphasis_NEL_TTP	0.78 (-0.58–0.97)
gldm_LowGrayLevelEmphasis_CET_TTP	0.67 (-1.32–0.95)
gldm_SmallDependenceEmphasis_CET_CBF	0.8 (-0.44–0.97)
gldm_SmallDependenceEmphasis_NEL_ADC	0.69 (-1.24–0.96)
gldm_SmallDependenceEmphasis_CET_ADC	0.96 (0.73–0.99)
gldm_SmallDependenceEmphasis_CET_CBV	0.85 (-0.04–0.98)
gldm_SmallDependenceHighGrayLevelEmphasis_CET_CBV	0.85 (-0.04–0.98)
gldm_SmallDependenceLowGrayLevelEmphasis_CET_CBF	0.84 (-0.12–0.98)
gldm_SmallDependenceLowGrayLevelEmphasis_NEL_OEF	0.94 (0.59–0.99)
gldm_SmallDependenceLowGrayLevelEmphasis_NEL_TTP	0.93 (0.48–0.99)
gldm_SmallDependenceLowGrayLevelEmphasis_CET_COV	0.84 (-0.15–0.98)
gldm_SmallDependenceLowGrayLevelEmphasis_CET_MTT	0.65 (-1.48–0.95)
gldm_SmallDependenceLowGrayLevelEmphasis_CET_OEF	0.8 (-0.42–0.97)
gldm_SmallDependenceLowGrayLevelEmphasis_CET_TTP	0.69 (-1.2–0.96)
gldm_SmallDependenceLowGrayLevelEmphasis_CET_ADC	0.61 (-1.77–0.95)
gldm_SmallDependenceLowGrayLevelEmphasis_CET_CBV	0.85 (-0.04–0.98)
gldm_SmallDependenceLowGrayLevelEmphasis_NEL_COV	0.85 (-0.08–0.98)
glrlm_GrayLevelNonUniformity_NEL_ADC	0.82 (-0.3–0.97)
glrlm_GrayLevelNonUniformity_NEL_CBF	0.77 (-0.67–0.97)
glrlm_GrayLevelNonUniformity_NEL_CBV	0.77 (-0.61–0.97)
glrlm_GrayLevelNonUniformityNormalized_NEL_TTP	0.54 (-2.28–0.94)
glrlm_GrayLevelVariance_NEL_ADC	0.81 (-0.38–0.97)
glrlm_GrayLevelVariance_CET_ADC	0.75 (-0.81–0.96)
glrlm_LongRunEmphasis_NEL_ADC	0.65 (-1.48–0.95)
glrlm_LongRunEmphasis_CET_ADC	0.95 (0.67–0.99)
glrlm_LongRunEmphasis_NEL_CBF	0.52 (-2.47–0.93)
glrlm_LongRunEmphasis_NEL_CBV	0.55 (-2.23–0.94)
glrlm_LongRunHighGrayLevelEmphasis_NEL_CBF	0.52 (-2.46–0.93)
glrlm_LongRunHighGrayLevelEmphasis_NEL_CBV	0.55 (-2.23–0.94)
glrlm_LongRunLowGrayLevelEmphasis_NEL_CBF	0.51 (-2.47–0.93)
glrlm_LongRunLowGrayLevelEmphasis_NEL_CBV	0.55 (-2.23–0.94)
glrlm_LowGrayLevelRunEmphasis_NEL_TTP	0.78 (-0.55–0.97)
glrlm_LowGrayLevelRunEmphasis_CET_TTP	0.71 (-1.09–0.96)
glrlm_RunEntropy_CET_CBF	0.81 (-0.38–0.97)
glszm_ZoneEntropy_NEL_CBF	0.64 (-0.03–0.87)
glszm_ZoneEntropy_NEL_CBV	0.56 (-0.26–0.85)
glszm_ZonePercentage_NEL_ADC	0.82 (0.49–0.94)
ngtdm_Busyness_NEL_TTP	0.68 (0.09–0.89)
ngtdm_Coarseness_NEL_CBV	0.67 (0.05–0.88)
ngtdm_Coarseness_CET_CBV	0.67 (0.05–0.88)
ngtdm_Contrast_NEL_OEF	0.56 (-0.26–0.85)
ngtdm_Strength_NEL_CMRO2	0.5 (-0.42–0.83)
CBV_BD	0.58 (-0.19–0.85)

glrlm_RunEntropy_NEL_TTP	0.65 (-1.51–0.95)
glrlm_RunEntropy_CET_CBV	0.75 (-0.81–0.96)
glrlm_RunLengthNonUniformity_CET_CBF	0.79 (-0.47–0.97)
glrlm_RunLengthNonUniformity_NEL_TTP	0.72 (-1.03–0.96)
glrlm_RunLengthNonUniformity_NEL_ADC	0.64 (-1.59–0.95)
glrlm_RunLengthNonUniformity_CET_COV	0.65 (-1.49–0.95)
glrlm_RunLengthNonUniformity_CET_CTH	0.64 (-1.54–0.95)
glrlm_RunLengthNonUniformity_CET_TTP	0.9 (0.26–0.99)
glrlm_RunLengthNonUniformity_NEL_CBF	0.84 (-0.12–0.98)
glrlm_RunLengthNonUniformity_NEL_CBV	0.83 (-0.22–0.98)
glrlm_RunLengthNonUniformity_CET_CBV	0.72 (-1.02–0.96)
glrlm_RunLengthNonUniformityNormalized_CET_CBF	0.75 (-0.81–0.96)
glrlm_RunLengthNonUniformityNormalized_NEL_ADC	0.56 (-2.15–0.94)
glrlm_RunLengthNonUniformityNormalized_CET_ADC	0.97 (0.79–1)
glrlm_RunLengthNonUniformityNormalized_CET_CBV	0.79 (-0.47–0.97)
glrlm_RunPercentage_CET_CBF	0.75 (-0.81–0.96)
glrlm_RunPercentage_NEL_ADC	0.59 (-1.93–0.94)
glrlm_RunPercentage_CET_ADC	0.97 (0.8–1)
glrlm_RunPercentage_CET_CBV	0.8 (-0.44–0.97)
glrlm_RunVariance_NEL_ADC	0.67 (-1.39–0.95)
glrlm_RunVariance_CET_ADC	0.93 (0.49–0.99)
glrlm_RunVariance_NEL_CBF	0.54 (-2.29–0.94)
glrlm_RunVariance_NEL_CBV	0.54 (-2.27–0.94)
glrlm_ShortRunEmphasis_CET_CBF	0.66 (-1.45–0.95)
glrlm_ShortRunEmphasis_NEL_ADC	0.59 (-1.96–0.94)
glrlm_ShortRunEmphasis_CET_ADC	0.97 (0.79–1)
glrlm_ShortRunEmphasis_NEL_CBF	0.7 (-1.11–0.96)
glrlm_ShortRunEmphasis_NEL_CBV	0.78 (-0.56–0.97)
glrlm_ShortRunEmphasis_CET_CBV	0.72 (-0.99–0.96)
glrlm_ShortRunHighGrayLevelEmphasis_NEL_CBV	0.78 (-0.56–0.97)
glrlm_ShortRunHighGrayLevelEmphasis_CET_CBV	0.72 (-0.99–0.96)
glrlm_ShortRunLowGrayLevelEmphasis_CET_CBF	0.72 (-0.99–0.96)
glrlm_ShortRunLowGrayLevelEmphasis_NEL_OEF	0.83 (-0.19–0.98)
glrlm_ShortRunLowGrayLevelEmphasis_NEL_TTP	0.85 (-0.1–0.98)
glrlm_ShortRunLowGrayLevelEmphasis_CET_TTP	0.66 (-1.44–0.95)
glrlm_ShortRunLowGrayLevelEmphasis_NEL_CBF	0.76 (-0.7–0.97)
glrlm_ShortRunLowGrayLevelEmphasis_NEL_CBV	0.78 (-0.56–0.97)
glrlm_ShortRunLowGrayLevelEmphasis_CET_CBV	0.72 (-0.99–0.96)
glrlm_ShortRunLowGrayLevelEmphasis_NEL_COV	0.82 (-0.26–0.98)
glszm_GrayLevelNonUniformity_CET_CBF	0.73 (-0.95–0.96)
glszm_GrayLevelNonUniformity_NEL_CMRO2	0.74 (-0.86–0.96)
glszm_GrayLevelNonUniformity_NEL_TTP	0.94 (0.59–0.99)
glszm_GrayLevelNonUniformity_NEL_ADC	0.72 (-1.02–0.96)
glszm_GrayLevelNonUniformity_CET_COV	0.94 (0.55–0.99)
glszm_GrayLevelNonUniformity_CET_CTH	0.89 (0.25–0.99)
glszm_GrayLevelNonUniformity_CET_MTT	0.53 (-2.37–0.93)
glszm_GrayLevelNonUniformity_CET_TTP	0.85 (-0.07–0.98)
glszm_GrayLevelNonUniformity_NEL_CBF	0.74 (-0.86–0.96)
glszm_GrayLevelNonUniformity_NEL_CBV	0.84 (-0.16–0.98)
glszm_GrayLevelNonUniformity_CET_CBV	0.69 (-1.18–0.96)
glszm_GrayLevelNonUniformityNormalized_NEL_TTP	0.73 (-0.95–0.96)
glszm_GrayLevelNonUniformityNormalized_NEL_ADC	0.61 (-1.8–0.95)
glszm_GrayLevelNonUniformityNormalized_CET_TTP	0.64 (-1.55–0.95)
glszm_GrayLevelVariance_NEL_ADC	0.55 (-2.19–0.94)
glszm_GrayLevelVariance_CET_ADC	0.81 (-0.36–0.97)
glszm_LargeAreaEmphasis_NEL_ADC	0.62 (-1.68–0.95)
glszm_LargeAreaEmphasis_CET_ADC	0.96 (0.75–1)
glszm_LargeAreaEmphasis_NEL_CBV	0.51 (-2.5–0.93)
glszm_LargeAreaHighGrayLevelEmphasis_CET_TTP	0.95 (0.65–0.99)
glszm_LargeAreaHighGrayLevelEmphasis_NEL_CBV	0.51 (-2.5–0.93)
glszm_LargeAreaLowGrayLevelEmphasis_NEL_TTP	0.63 (-1.62–0.95)
glszm_LargeAreaLowGrayLevelEmphasis_CET_TTP	0.51 (-2.51–0.93)
glszm_LargeAreaLowGrayLevelEmphasis_NEL_CBV	0.51 (-2.5–0.93)
glszm_LowGrayLevelZoneEmphasis_NEL_TTP	0.82 (-0.3–0.97)
glszm_LowGrayLevelZoneEmphasis_CET_TTP	0.55 (-2.22–0.94)
glszm_SizeZoneNonUniformityNormalized_NEL_OEF	0.55 (-2.23–0.94)
glszm_SizeZoneNonUniformityNormalized_NEL_ADC	0.88 (0.14–0.98)
glszm_SizeZoneNonUniformityNormalized_CET_ADC	0.92 (0.4–0.99)
glszm_SizeZoneNonUniformityNormalized_NEL_CBF	0.73 (-0.96–0.96)
glszm_SizeZoneNonUniformityNormalized_NEL_CBV	0.73 (-0.91–0.96)
glszm_SmallAreaEmphasis_NEL_ADC	0.89 (0.2–0.98)
glszm_SmallAreaEmphasis_CET_OEF	0.64 (-1.58–0.95)
glszm_SmallAreaEmphasis_CET_TTP	0.59 (-1.92–0.94)
glszm_SmallAreaEmphasis_CET_ADC	0.9 (0.31–0.99)
glszm_SmallAreaLowGrayLevelEmphasis_NEL_TTP	0.92 (0.45–0.99)
glszm_SmallAreaLowGrayLevelEmphasis_CET_ADC	0.64 (-1.54–0.95)
glszm_SmallAreaLowGrayLevelEmphasis_NEL_COV	0.66 (-1.45–0.95)
glszm_SmallAreaLowGrayLevelEmphasis_NEL_CTH	0.72 (-0.98–0.96)
glszm_ZoneEntropy_CET_CBF	0.69 (-1.21–0.96)
glszm_ZoneEntropy_NEL_TTP	0.84 (-0.16–0.98)
glszm_ZoneEntropy_NEL_ADC	0.62 (-1.69–0.95)
glszm_ZoneEntropy_CET_TTP	0.74 (-0.84–0.96)
glszm_ZoneEntropy_CET_ADC	0.5 (-2.56–0.93)
glszm_ZoneEntropy_NEL_CBF	0.85 (-0.05–0.98)
glszm_ZoneEntropy_NEL_CBV	0.84 (-0.14–0.98)
glszm_ZonePercentage_NEL_ADC	0.66 (-1.41–0.95)
glszm_ZonePercentage_CET_ADC	0.97 (0.78–1)
glszm_ZonePercentage_CET_CBV	0.63 (-1.62–0.95)
glszm_ZoneVariance_NEL_ADC	0.59 (-1.9–0.94)
glszm_ZoneVariance_CET_ADC	0.95 (0.67–0.99)
glszm_ZoneVariance_NEL_CBV	0.72 (-0.97–0.96)
ngtdm_Busyness_NEL_ADC	0.52 (-2.41–0.93)
ngtdm_Busyness_CET_ADC	0.56 (-2.18–0.94)

ngtdm_Coarseness_NEL_CMRO2	0.56 (-2.15–0.94)
ngtdm_Coarseness_CET_CMRO2	0.92 (0.4–0.99)
ngtdm_Coarseness_NEL_CBV	0.67 (-1.37–0.95)
ngtdm_Coarseness_CET_CBV	0.67 (-1.37–0.95)
ngtdm_Contrast_NEL_ADC	0.72 (-0.98–0.96)
CTH_Intersection	0.5 (-2.57–0.93)
